# A PI3K p110β–Rac signalling loop mediates Pten-loss-induced perturbation of haematopoiesis and leukaemogenesis

**DOI:** 10.1038/ncomms9501

**Published:** 2015-10-07

**Authors:** Haluk Yuzugullu, Lukas Baitsch, Thanh Von, Allison Steiner, Haoxuan Tong, Jing Ni, Linda K. Clayton, Roderick Bronson, Thomas M. Roberts, Kira Gritsman, Jean J. Zhao

**Affiliations:** 1 Department of Cancer Biology, Dana-Farber Cancer Institute, Boston, Massachusetts 02215, USA; 2 Department of Biological Chemistry and Molecular Pharmacology, Harvard Medical School, Boston, Massachusetts 02215, USA; 3 Dana-Farber/Harvard Cancer Center Rodent Histopathology Core, Harvard Medical School, Boston, Massachusetts 02215, USA; 4 Present address: Department of Medicine, Department of Cell Biology, Albert Einstein College of Medicine, Bronx, New York 10461, USA

## Abstract

The tumour suppressor PTEN, which antagonizes PI3K signalling, is frequently inactivated in haematologic malignancies. In mice, deletion of PTEN in haematopoietic stem cells (HSCs) causes perturbed haematopoiesis, myeloproliferative neoplasia (MPN) and leukaemia. Although the roles of the PI3K isoforms have been studied in PTEN-deficient tumours, their individual roles in PTEN-deficient HSCs are unknown. Here we show that when we delete PTEN in HSCs using the Mx1–Cre system, p110β ablation prevents MPN, improves HSC function and suppresses leukaemia initiation. Pharmacologic inhibition of p110β in PTEN-deficient mice recapitulates these genetic findings, but suggests involvement of both Akt-dependent and -independent pathways. Further investigation reveals that a p110β–Rac signalling loop plays a critical role in PTEN-deficient HSCs. Together, these data suggest that myeloid neoplasia driven by PTEN loss is dependent on p110β via p110β–Rac-positive-feedback loop, and that disruption of this loop may offer a new and effective therapeutic strategy for PTEN-deficient leukaemia.

## Abstract (web summary)

The tumor suppressor PTEN antagonizes the PI3K signalling pathway and is frequently inactivated in haematological malignancies. Here, the authors unravel the main contribution of the PI3K isoform p110ß to leukemic transformation driven by PTEN-loss.

## Graphical abstract

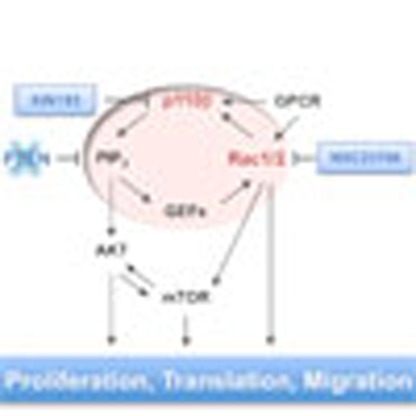

Dysregulation of the molecular pathways involved in the self-renewal, differentiation and proliferation of haematopoietic stem cells (HSCs) can cause leukaemia. Notably, the serine/threonine kinase Akt, which acts downstream of PI3 kinase (PI3K), is hyper-phosphorylated in up to 80% of acute myeloid leukaemia (AML) cases^1^. This is unlikely to be due to mutations in upstream receptor tyrosine kinases alone. In chronic myelogenous leukaemia, PI3K/Akt signalling can also be activated through downregulation of the phosphatase and tensin homologue (PTEN) by BCR–ABL^2^. PTEN is a lipid phosphatase that counteracts PI3K signalling by dephosphorylating phosphatidylinositol-3,4,5-trisphosphate (PIP3). PTEN is frequently inactivated in haematological malignancies^3^,^4^, including in AML and T cell acute lymphoblastic leukemia (T-ALL)^5^. Notably, PTEN expression is often reduced in the disease through several other modes of PTEN regulation, for example, microRNAs, epigenetic modifications and ubiquitination^6^,^7^,^8^,^9^, which likely contribute to the high frequency of Akt phosphorylation in myeloid leukaemia.

In mice, genetic ablation of PTEN in the haematopoietic system leads to HSC depletion in the bone marrow (BM), myeloproliferative neoplasia (MPN) and transplantable acute leukaemia (myeloid or T-cell leukaemia)^10^,^11^,^12^. In patients, MPNs such as chronic myelogenous leukaemia or myelofibrosis can progress to AML^13^.

Class I PI3Ks are heterodimeric lipid kinases that produce the lipid second messenger PIP3 on stimulation of cells by many growth factors. Class I PI3Ks are divided into class IA (p110α, p110β and p110δ) and class IB (p110γ) enzymes; of these, the p110α and p110β isoforms are ubiquitously expressed, while p110δ and p110γ are enriched in leukocytes. Work in several different murine models has documented distinct requirements for different PI3K isoforms in particular tumour types^14^,^15^. For example, p110α is essential in a model of mutant Kras-induced lung adenocarcinoma^16^. Recently, we showed that Ras-mutated myeloid leukaemia is also dependent on the p110α isoform, and combined pharmacologic inhibition of p110α and mitogen-activated protein kinase kinase (MEK) could be an effective therapeutic strategy for Ras-mutated myeloid malignancies^17^. Although p110β plays a less prominent role in receptor tyrosine kinase (RTK) signalling, it mediates G protein-coupled receptor (GPCR) and integrin signalling^18^,^19^,^20^, and has been shown to interact specifically with Rho family GTPases Rac1 and CDC42 (ref. 21). Several recent studies demonstrated that p110β is required in many, but not all, PTEN-deficient solid tumours^20^,^22^,^23^. However, it is not known which PI3K isoforms are most important for myeloid neoplastic transformation driven by PTEN loss.

A number of pan-class I PI3K and dual class I/mTOR inhibitors are now in clinical trials for cancer, including leukaemia. However, targeting PI3K with these inhibitors could potentially lead to severe toxicity, which could be prevented by targeting single PI3K isoforms. To this end, numerous isoform-selective compounds are currently under development with some already in clinical trials^14^. The p110δ-selective inhibitor idelalisib (referred to here as GS1101) has been remarkably effective in treating indolent B-cell malignancies, and is now approved by the FDA for the treatment of chronic lymphocytic leukaemia^24^. In the case of solid tumours, p110α-selective inhibitors have shown great promise in early-phase trials for patients with tumours bearing *PIK3CA* mutations^14^. Notably, selective inhibitors of p110β are in clinical trials as anticancer reagents for advanced solid tumours with PTEN deficiency (NCT01458067). Thus, unravelling the role of each PI3K isoform, and its contribution to leukaemic transformation driven by PTEN loss, would inform rational approaches in targeting the PI3K pathway with a better therapeutic window.

In the present study, we used genetically engineered mouse models to determine which of the class IA PI3K isoforms are most important in mediating the effects of Pten loss in HSCs. We show that, in the setting of Pten loss, p110β is the main PI3K isoform responsible for MPN development and HSC depletion in the BM. Furthermore, we show that isoform-selective PI3K inhibitors recapitulate our genetic findings. We also found that a signalling loop featuring p110β and Rac plays an important role in the absence of Pten. Our results suggest that targeting p110β and/or Rac may lead to an effective therapeutic strategy for PTEN-deficient myeloid leukaemia.

## Results

### p110β ablation prolongs survival of Mx-1-Cre;Pten^fl/fl^ mice

To explore the roles of the class IA PI3K isoforms in HSCs and leukaemic transformation in the absence of Pten, we crossed Mx-1-Cre^+^;Pten^fl/fl^ mice^10^,^11^ with strains that carry homozygous floxed alleles of p110α (ref. 25) or p110β (ref. 20), or with a p110δ germline knockout strain^26^ to allow for simultaneous deletion of Pten and PI3K isoforms in HSCs. As Mx1 is an interferon-responsive promoter expressed in HSCs and all other haematopoietic cells, the expression of Cre recombinase can be induced by administration of double-stranded RNA polyI-polyC (pIpC), which induces an interferon response^10^,^27^. Mx-1-Cre^+^;Pten^fl/fl^ (*Pten*^*Δ/Δ*^), Mx-1-Cre^+^;Pten^fl/fl^;p110α^fl/fl^ (*Pten*^*Δ/Δ*^*;p110α*^*Δ/Δ*^), Mx-1-Cre^+^;Pten^fl/fl^;p110β^fl/fl^ (*Pten*^*Δ/Δ*^*;p110β*^*Δ/Δ*^) and Mx-1-Cre^+^;Pten^fl/fl^;p110δ^KO/KO^ (*Pten*^*Δ/Δ*^*;p110δ*^*−/−*^) mice were treated with pIpC to induce Cre-mediated excision of floxed alleles at 4 weeks of age (Supplementary Fig. 1a). This leads to the deletion of Pten and/or PI3K isoforms in haematopoietic cells, including HSCs or progenitor cells in the BM, spleen, thymus and liver^11^.

**Figure 1:**
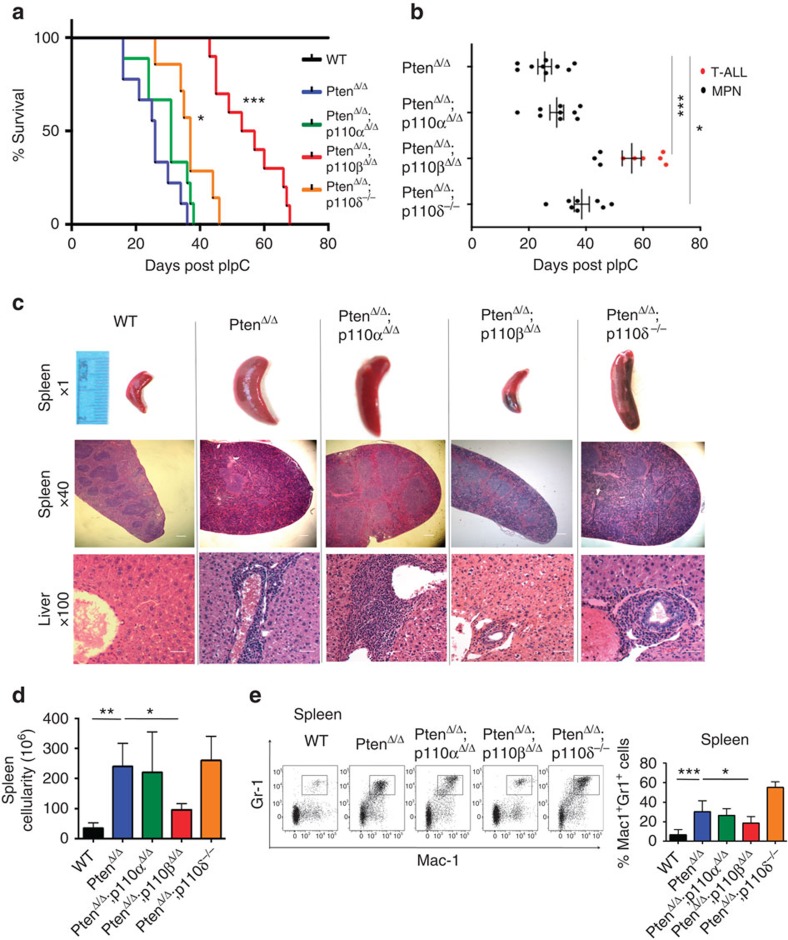
Ablation of p110β prolonged survival of animals on Pten loss. (**a**) Survival of *Pten*^*Δ/Δ*^ (blue; *n*=9; median survival=26 days), *Pten*^*Δ/Δ*^*;p110α*^*Δ/Δ*^ (green; *n*=9; median survival=31 days), *Pten*^*Δ/Δ*^*;p110β*^*Δ/Δ*^ (red; *n*=9; median survival=55 days), *Pten*^*Δ/Δ*^*;p110δ*^*−/−*^ (orange; *n*=7; median survival=37 days) and control animals (black) represented as DPI. The log-rank test was used to compare survival between groups. (**b**) Survival time and the development of MPN and T-ALL in each group of mice as indicated. Survival of *Pten*^*Δ/Δ*^ (*n*=9; median survival=26 days), *Pten*^*Δ/Δ*^*;p110α*^*Δ/Δ*^ (*n*=9; median survival=31 days), *Pten*^*Δ/Δ*^*;p110β*^*Δ/Δ*^ (*n*=9; median survival=55 days), *Pten*^*Δ/Δ*^*;p110δ*^*−/−*^ (*n*=7; median survival=37 days) and control animals (black) represented as DPI. Black dots represent MPN disease cases and red dots represent T-ALL cases in each group. The log-rank test was used to compare survival between groups. (**c**) Histopathology of the spleen (× 1 and × 40) and liver (× 100) taken at 26 DPI. Increased splenomegaly and disruption of spleen architecture as well as increased myeloid cell infiltration after Pten deletion. Scale bar, 5 μm. (**d**) Total cellularity of the spleen is represented as mean±s.d. of *n*=5 except *Pten*^*Δ/Δ*^*;p110δ*^*−/−*^ (*n*=10). Two-way ANOVA test was applied to compare the spleen cellularity. (**e**) Representative flow cytometry plots of Mac1^+^Gr1^+^ cells in the spleen. Samples were analysed at 26 DPI. Bar graphs represent the mean±s.d. of *n*=9 (*Pten*^*Δ/Δ*^*;p110δ*^*−/−*^), 10 (*Pten*^*Δ/Δ*^*;p110α*^*Δ/Δ*^), 11 (ctrl) or 13 (*Pten*^*Δ/Δ*^ and *Pten*^*Δ/Δ*^*;p110β*^*Δ/Δ*^). Two-way ANOVA test was applied to compare the Mac1-Gr1 cells in the spleen. *P<0.05, **P<0.01, ***P<0.001

Consistent with previous studies^10^,^11^, all *Pten*^*Δ/Δ*^ mice developed MPN and reached the survival end point 20–40 days post injection (DPI; Fig. 1a). *Pten*^*Δ/Δ*^*;p110α*^*Δ/Δ*^ and *Pten*^*Δ/Δ*^*;p110δ*^*−/−*^ mice also developed MPN with slightly extended survival (Fig. 1a). Notably, *Pten*^*Δ/Δ*^*;p110β*^*Δ/Δ*^ mice lived the longest, with median survival significantly longer than that of any other group (Fig. 1a). Further observation revealed that, whereas control, *Pten*^*Δ/Δ*^, *Pten*^*Δ/Δ*^*;p110α*^*Δ/Δ*^ and *Pten*^*Δ/Δ*^*;p110δ*^*−/−*^ animals developed MPN, six out of nine *Pten*^*Δ/Δ*^*;p110β*^*Δ/Δ*^ mice succumbed to T-ALL (Fig. 1b). BM from the three *Pten*^*Δ/Δ*^*;p110β*^*Δ/Δ*^ mice that did develop MPN was analysed for excision of the pik3cb allele. Notably, we found that BM of these mice had incomplete deletion of Pik3cb alleles suggesting that p110β is critical for the development of MPN in this model (Supplementary Fig. 1b). Deletion of p110β in HSCs using Mx1–Cre in animals that are wild-type (WT) for Pten does not significantly affect blood counts (Supplementary Fig. 1c).

Histopathological analysis of moribund animals of *Pten*^*Δ/Δ*^, *Pten*^*Δ/Δ*^*;p110α*^*Δ/Δ*^ and *Pten*^*Δ/Δ*^*;p110δ*^*−/−*^ mice at 20–40 days post pIpC showed that they developed massive splenomegaly with a marked increase in cells expressing myeloperoxidase, a marker used to detect leukaemic cells of the myeloid lineage, in both the spleen and liver (Supplementary Fig. 2a), confirming MPN development in these mice. Notably, thymuses in these moribund mice appeared normal (Supplementary Fig. 2b). However, most *Pten*^*Δ/Δ*^*;p110β*^*Δ/Δ*^ animals became moribund at 50–70 days post pIpC with markedly increased thymus weights (Supplementary Fig. 2b), infiltration of terminal deoxynucleotidyl transferase-positive T lymphoblasts (CD4^+^ or CD4 and CD8 double-positive T-cell blasts) in the thymus and BM and increased white blood cell counts (Supplementary Fig. 2a,c–e), all of which are manifestations of T-ALL. These results suggest that the p110β isoform of PI3K plays a uniquely important role in driving myeloid neoplastic transformation in mice with Pten-deficient HSCs, but does not contribute to the development of T-ALL in this murine model.

Previous studies have shown that Pten deletion in T-cell progenitors causes malignant transformation in the thymus and leads to T-cell lymphoma/T-ALL within 50–150 days^28^,^29^. Mice with Mx1–Cre-mediated deletion of *Pten*^*Δ/Δ*^, *Pten*^*Δ/Δ*^*;p110α*^*Δ/Δ*^ or *Pten*^*Δ/Δ*^*;p110δ*^*−/−*^ showed infiltrating MPN disease and became moribund within 20–40 days after pIpC, earlier than the disease latency for T-cell disease development. Since *Pten*^*Δ/Δ*^*;p110β*^*Δ/Δ*^ mice did not develop MPN, they survived longer and developed T-cell lymphoma/T-ALL ∼50–70 days post pIpC injection, a timeline consistent with previous reports on T-cell lymphoma/T-ALL formation in models of Pten loss in T-cell progenitors^28^,^29^. This suggests that p110β ablation does not prevent T-ALL formation driven by Pten loss.

To examine further the PI3K isoform dependence in T-ALL induced by Pten loss, we investigated the roles of p110α and p110β in a different T-ALL model driven by Pten ablation in T-cell progenitors using Lck–Cre^30^. Interestingly, deletion of either p110α or p110β had no effect on T-ALL in this model 3 (Supplementary Fig. 2f). These results underscore the distinct roles of p110β in myeloid and lymphoid neoplasia induced by Pten deletion in haematopoietic cells.

### p110β mediates myeloid expansion induced by Pten loss

To further characterize disease in the *Pten*^*Δ/Δ*^*;p110*^*Δ/Δ*^ mice, we killed mice of each genotype at 26 DPI, the time point at which the *Pten*^*Δ/Δ*^ mice become moribund. Similar to previous reports, all animals in the *Pten*^*Δ/Δ*^ group displayed massive splenomegaly, increased spleen cellularity and loss of spleen architecture at this time point (Fig. 1c,d). Both *Pten*^*Δ/Δ*^*;p110α*^*Δ/Δ*^ and *Pten*^*Δ/Δ*^*;p110δ*^*−/−*^ mice showed evidence of MPN similar to that of *Pten*^*Δ/Δ*^ mice, suggesting that ablation of p110α or p110δ failed to rescue this disease phenotype. Notably, *Pten*^*Δ/Δ*^*;p110β*^*Δ/Δ*^ mice had significantly reduced spleen cellularity and size, compared with *Pten*^*Δ/Δ*^ mice (Fig. 1c,d). Consistently, pathological analysis of the spleen and liver revealed infiltration of myeloid cells in *Pten*^*Δ/Δ*^, *Pten*^*Δ/Δ*^*;p110α*^*Δ/Δ*^ and *Pten*^*Δ/Δ*^*;p110δ*^*−/−*^ animals, but not in *Pten*^*Δ/Δ*^*;p110β*^*Δ/Δ*^ mice (Fig. 1c). Flow cytometric analysis confirmed an increased population of myeloid cells (Mac1^+^Gr1^+^) in the BM, spleen and peripheral blood of *Pten*^*Δ/Δ*^ animals (Fig. 1e; Supplementary Fig. 3). Again, the numbers of Mac1^+^Gr1^+^ cells in these organs in *Pten*^*Δ/Δ*^*;p110β*^*Δ/Δ*^ but not in *Pten*^*Δ/Δ*^*;p110α*^*Δ/Δ*^ and *Pten*^*Δ/Δ*^*;p110δ*^*−/−*^ animals were consistently reduced compared with those of *Pten*^*Δ/Δ*^ mice (Fig. 1e; Supplementary Fig. 3), suggesting that ablation of p110β suppressed myeloid cell expansion on Pten loss.

**Figure 2:**
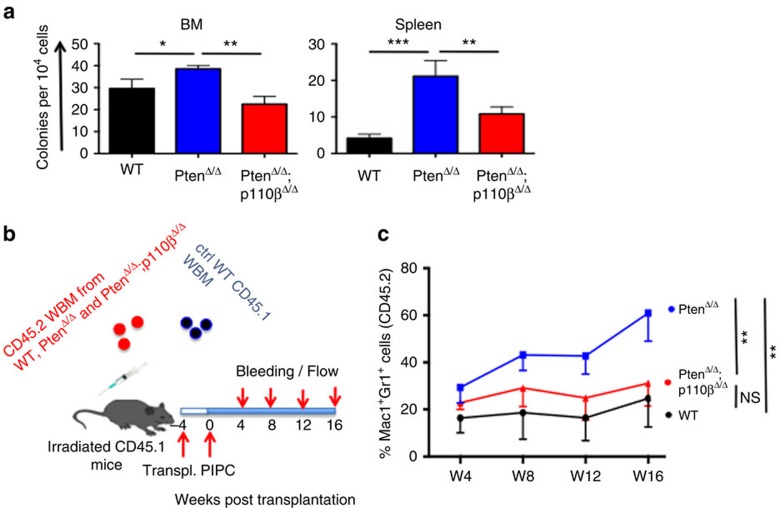
Ablation of p110β blocked MPN development in a cell-autonomous manner. (**a**) Colony-forming assays were performed with 1 × 10^4^ (BM) or 5 × 10^4^ (spleen) cells obtained from corresponding animals. Cells were cultured in M3434 medium. Colonies were counted 7 days after incubation. Data are represented as mean±s.e.m. (*n*=3, in duplicates). *P<0.05, **P<0.01, ***P<0.001. (**b**) BM cells were isolated from CD45.2^+^ WT, *Pten*^*Δ/Δ*^ and *Pten*^*Δ/Δ*^*;p110β*^*Δ/Δ*^ animals and transplanted into CD45.1^+^ mice as shown in the figure (*n*=10 for each group). (**c**) The myeloid cell reconstitution potential of the CD45.2^+^ cells was measured at regular intervals for 16 weeks. WT and *Pten*^*Δ/Δ*^*;p110β*^*Δ/Δ*^-derived myeloid cells have a stable amount of CD45.2^+^ myeloid cells; however, *Pten*^*Δ/Δ*^-derived myeloid cells expanded significantly and reached to 60% at week 16 (*n*=10 for all groups, **P<0.001). Two-way ANOVA test was applied to compare the myeloid cell potential.

To further validate the role of p110β in the myeloid expansion caused by Pten loss, we performed colony assays in methylcellulose supplemented with myeloid growth factors. Compared with wild-type controls, both BM and spleen cells from *Pten*^*Δ/Δ*^ animals generated an increased number of colonies, which was significantly reduced in *Pten*^*Δ/Δ*^*;p110β*^*Δ/Δ*^ mice (Fig. 2a). Together, these results demonstrate that p110β is required for MPN development in the absence of Pten.

To determine whether the contribution of p110β to myeloid neoplasia in the absence of Pten is a cell-autonomous or indirect effect, we transplanted whole BM cells from *Pten*^*Δ/Δ*^, *Pten*^*Δ/Δ*^*;p110β*^*Δ/Δ*^ or control mice into recipient mice (Fig. 2b). Four weeks after transplantation, all groups were treated with pIpC, and the relative frequency of CD45.2^+^ donor-derived Mac1^+^Gr1^+^ cells was monitored over 16 weeks. The proportion of *Pten*^*Δ/Δ*^ donor-derived myeloid cells expanded significantly 4 weeks after pIpC and remained elevated during the course of the experiment. In contrast, the *Pten*^*Δ/Δ*^*;p110β*^*Δ/Δ*^ donor-derived myeloid population remained stable during the entire experiment, with levels much comparable to that of wild-type control mice (Fig. 2c). There were no significant differences in the percentage of donor-derived CD3-positive T cells among any of the groups tested (Supplementary Fig. 4a). The percentage of donor-derived B220-positive B cells was reduced after Pten deletion, and deletion of p110β did not alter B-cell chimaerism (Supplementary Fig. 4b). These data suggest that p110β mediates the expansion of myeloid cells in a cell-autonomous manner.

### p110β perturbs HSC homeostasis on loss of Pten

**Figure 3:**
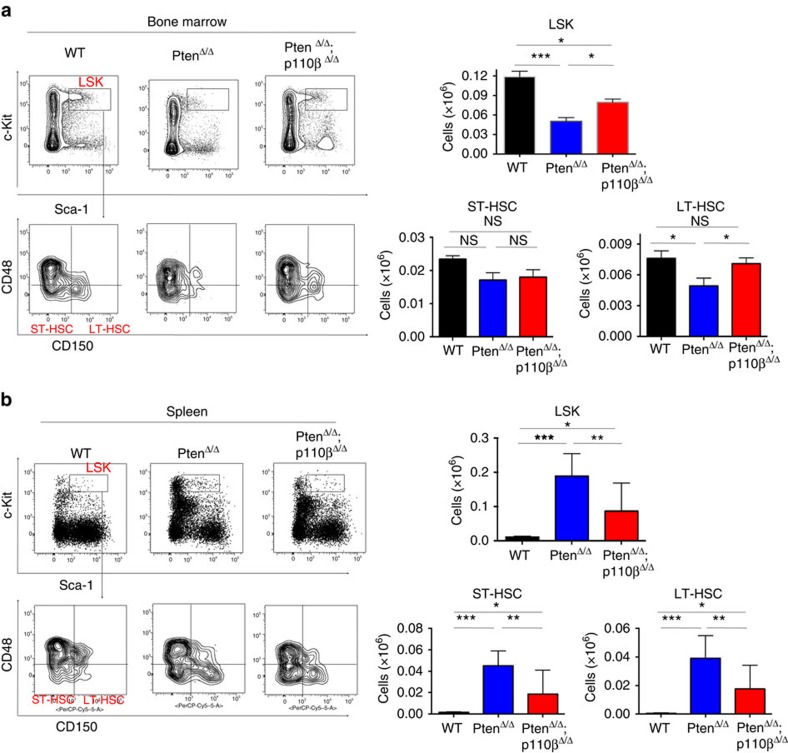
p110β deletion restores HSCs to both the bone marrow (BM) and the spleen. (**a**) Representative flow cytometric plots of ctrl, *Pten*^*Δ/Δ*^ and *Pten*^*Δ/Δ*^*;p110β*^*Δ/Δ*^ BM showing LSK cells (Lin^–^Sca-1^+^c-kit^+^), ST-HSC (CD150^–^CD48^−^Lin^−^Sca-1^+^c-kit^+^) and LT-HSC (CD150^+^CD48^−^Lin^−^Sca-1^+^c-kit^+^) cells (left panels) gated on total living CD45.2^+^Lin^–^ cells. Total counts LSK (top) and LT-HSC (bottom right) of lineage-negative cells in the BM measured at 26 DPI in ctrl (*n*=13), *Pten*^*Δ/Δ*^ (*n*=12) and *Pten*^*Δ/Δ*^*;p110β*^*Δ/Δ*^ (*n*=18) animals. Two-way ANOVA test was applied to compare the stem cell numbers. (**b**) Representative flow cytometry plots of control, *Pten*^*Δ/Δ*^ and *Pten*^*Δ/Δ*^*;p110β*^*Δ/Δ*^ spleen cells showing LSK cells (Lin^–^Sca-1^+^c-kit^+^), ST-HSC (CD150^–^CD48^−^Lin^−^Sca-1^+^c-kit^+^) and LT-HSC (CD150^+^CD48^−^Lin^−^Sca-1^+^c-kit^+^) cells (left panels). Total LSK (top) and ST-HSC and LT-HSC (bottom right) cells in the spleen measured at 26 DPI. Numbers represent total cell counts per spleen (*n*=7 (ctrl, *Pten*^*Δ/Δ*^) and 10 (*Pten*^*Δ/Δ*^*;p110β*^*Δ/Δ*^)). Two-way ANOVA test was applied to compare the stem cell numbers. *P<0.05, **P<0.01, ***P<0.001

Earlier studies showed that HSC-specific deletion of Pten leads to the exhaustion of HSCs in the BM, their accumulation in the periphery and extramedullary haematopoiesis^10^,^11^. Hence, we wanted to test whether p110β ablation could rescue HSCs. In *Pten*^*Δ/Δ*^ mice, the numbers of both the Lin^−^Sca-1^+^c-kit^+^ (LSK) cells, containing HSCs and the CD150^+^CD48^−^Lin^−^Sca-1^+^c-kit^+^ population, which is enriched for long-term HSCs (LT-HSCs) were significantly reduced at 26 DPI, consistent with previous findings^11^ (Fig. 3a; Supplementary Fig. 5a). Ablation of p110β was able to partially rescue LSK cells and restore LT-HSCs in Pten-null BM (Fig. 3a). Loss of Pten did not change the total number of myeloid progenitors, or the frequencies of the common myeloid progenitors and granulocyte macrophage progenitors or megakaryocyte-erythroid progenitors, but led to a significant decrease in the number of common lymphoid progenitors consistent with original reports^11^ (Supplementary Fig. 5b). This decrease in the number of common lymphoid progenitors in the Pten-deficient BM was not rescued by the deletion of p110β (Supplementary Fig. 5b).

To determine whether p110β is responsible for the increased extramedullary haematopoiesis in the spleen seen after Pten loss, we measured the frequency and absolute numbers of HSCs and progenitors in the spleens of *Pten*^*Δ/Δ*^ and *Pten*^*Δ/Δ*^*;p110β*^*Δ/Δ*^ animals. Pten deficiency led to a significant increase in the number of LSK cells, as well as LT-HSCs, short-term HSCs (ST-HSCs) and progenitors in the spleens of *Pten*^*Δ/Δ*^ animals (Fig. 3b; Supplementary Fig. 5c,d)^11^,^31^, which was partially suppressed in the spleens of *Pten*^*Δ/Δ*^*;p110β*^*Δ/Δ*^ animals (Fig. 3b; Supplementary Fig. 5c,d). These results suggest that deletion of p110β normalizes the distribution of phenotypic LSK cells between the BM and extramedullary tissues in *Pten*^*Δ/Δ*^*;p110β*^*Δ/Δ*^ animals compared with *Pten*^*Δ/Δ*^ controls. Thus, our findings are consistent with the idea that p110β contributes to the perturbed HSC homeostasis observed in the absence of Pten leading to extramedullary haematopoiesis and the development of MPN.

### p110β mediates leukaemia initiation in the absence of Pten

**Figure 4:**
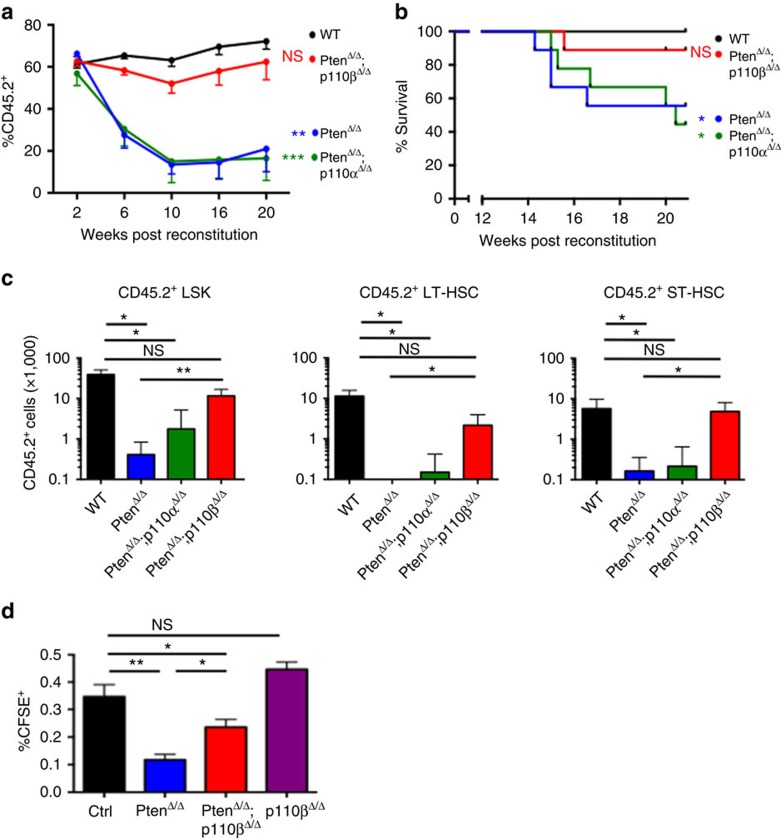
p110β deletion rescued long-term HSC (LT-HSC) reconstitution and suppressed leukaemia initiation and homing defects after Pten deletion. (**a**) Competitive repopulation experiment: bone marrow (BM) cells were isolated at 7 days post pIpC (DPI) from CD45.2^+^ ctrl, *Pten*^*Δ/Δ*^, *Pten*^*Δ/Δ*^*;p110α*^*Δ/Δ*^ and *Pten*^*Δ/Δ*^*;p110β*^*Δ/Δ*^ animals, and transplanted into lethally irradiated recipient animals along with CD45.1^+^ BM cells. The donor chimaerism in the peripheral blood was measured at regular intervals for 20 weeks, represented as the percentage of CD45.2^+^ cells among all leukocytes in the peripheral blood (*n*=9 for all groups). Two-way ANOVA test was applied to compare the donor derived cell numbers. (**b**) Disease-free survival of animals in the competitive repopulation experiment. Statistical analysis was performed at week 20 post transplantation using Fisher’s exact test. All samples were compared with the control group. Fischer exact test was applied to test overall survival. (**c**) After 20 weeks, the competitive repopulation experiment was terminated, and the remaining numbers of LSK, ST-HSC and LT-HSCs in the BM were measured by flow cytometry (*n*=4 for each group except *n*=5 for *Pten*^*Δ/Δ*^*;p110δ*^*−/−*^). Two-way ANOVA test was applied to compare the donor derived stem cell numbers. (**d**) Homing potential of CD45.2-positive carboxyfluorescein diacetate succinimidyl ester (CFSE)-labelled control, *Pten*^*Δ/Δ*^ and *Pten*^*Δ/Δ*^*;p110β*^*Δ/Δ*^ cells in 24 h in irradiated recipients (*n*=5) for each group. Two-way ANOVA test was applied to compare the donor derived stem cell numbers. *P<0.05, **P< 0.01, ***P<0.001

It has been reported that Pten loss in the BM leads to the depletion of HSCs and the generation of leukaemia-initiating cells^10^,^11^. To determine whether the p110β isoform also uniquely plays critical roles in these processes in the Pten-loss setting, we performed competitive multi-lineage repopulation assays to compare the contribution of marked (CD45.2) *Pten*^*Δ/Δ*^, *Pten*^*Δ/Δ*^*;p110α*^*Δ/Δ*^, *Pten*^*Δ/Δ*^*;p110β*^*Δ/Δ*^ donor BM cells to that of wild-type competitor cells (CD45.1) following transplantation into lethally irradiated mice. Consistent with earlier reports, the contribution of donor-derived cells to the peripheral blood was progressively decreased and eventually depleted for *Pten*^*Δ/Δ*^ donors, but not for control donors^10^,^11^ (Fig. 4a). HSCs derived from *Pten*^*Δ/Δ*^*;p110α*^*Δ/Δ*^ mice also failed to sustain long-term reconstitution. In striking contrast, HSCs derived from *Pten*^*Δ/Δ*^*;p110β*^*Δ/Δ*^ mice were able to reconstitute recipient animals for more than 20 weeks (Fig. 4a). In addition, the majority of *Pten*^*Δ/Δ*^ and *Pten*^*Δ/Δ*^*;p110α*^*Δ/Δ*^-recipient mice developed T-ALL as evidenced by the abundance of donor-derived CD45.2^+^ CD3^+^;CD4^+^ or CD3^+^CD4^−^ T lymphoblasts at the experimental end point of 20 weeks in these mice (Fig. 4b; Supplementary Fig. 6). In contrast, recipients of BM from wild-type control mice and from the majority of *Pten*^*Δ/Δ*^*;p110β*^*Δ/Δ*^ animals remained leukaemia free with few CD45.2^+^CD3^+^ cells at the experimental end point (Fig. 4b; Supplementary Fig. 6). Furthermore, analysis of the BM at week 20 showed that the donor chimaerism in the LSK, ST-HSC and LT-HSC compartments was significantly improved in the *Pten*^*Δ/Δ*^ and *Pten*^*Δ/Δ*^*;p110α*^*Δ/Δ*^ groups (Fig. 4c). These data suggest that, in the absence of Pten, p110β is the major PI3K isoform critical for the loss of HSCs and for leukaemia initiation.

To determine the cellular mechanism underlying the improved reconstitution of Pten-null BM cells on loss of p110β, we examined the cell cycle status, senescence, apoptosis and homing properties of HSCs. As reported earlier, we also found that loss of Pten led to increased cycling of HSCs and reduced the number of quiescent HSCs (Supplementary Fig. 7a)^11^. Although ablation of p110β resulted in a tendency towards rescuing these effects, the results did not reach statistical significance (Supplementary Fig. 7a). Moreover, we did not observe any change in the proportion of whole BM or LSK cells expressing senescence-associated β-gal activity or undergoing apoptosis in any of the groups tested (Supplementary Fig. 7b,c). We then performed homing assays, in which fluorescently labelled BM cells were transplanted into irradiated wild-type hosts, and donor-derived cells were quantified after 24 h. We found that Pten deficiency significantly reduced the homing capacity of transplanted cells to the BM, and p110β ablation could partially rescue the homing potential (Fig. 4d). We also found that p110β ablation does not affect homing in Pten-wild-type BM cells (Fig. 4d). Thus, we conclude that p110β is responsible, at least in part, for the reduced homing activity of Pten-deficient HSCs.

### Inhibition of p110β suppresses myeloid leukaemogenesis

**Figure 5:**
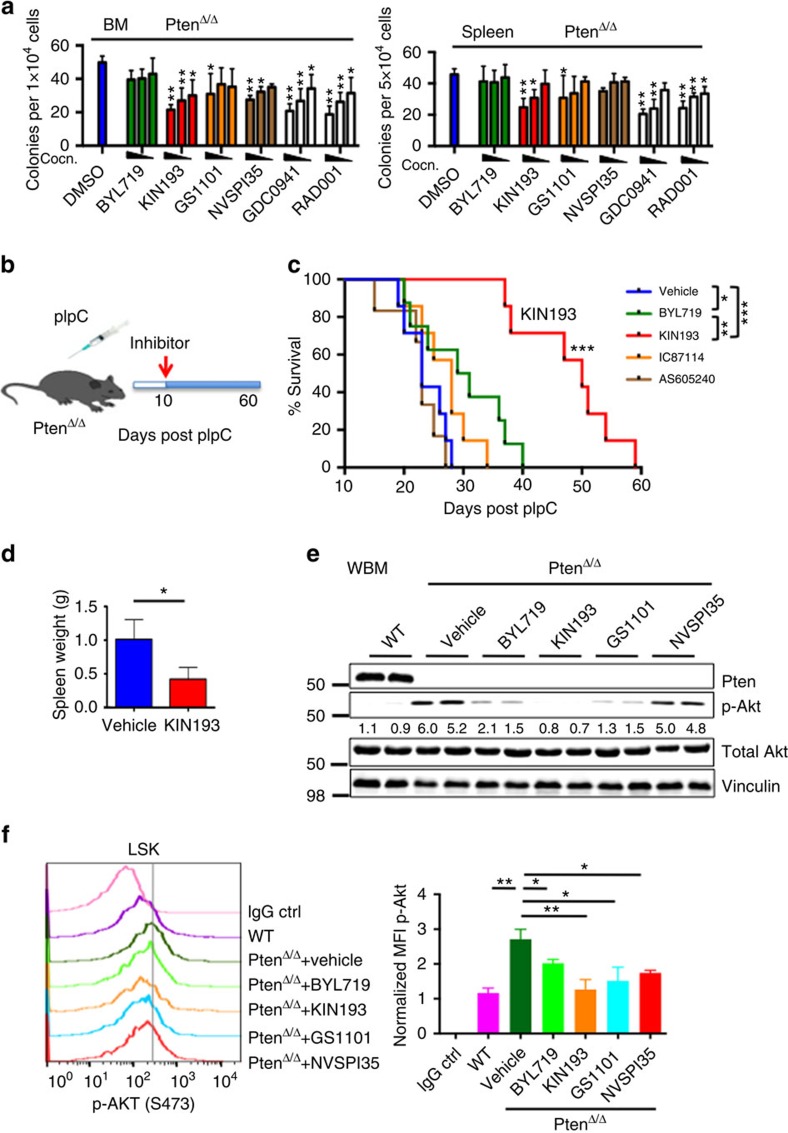
Pharmacological inhibition of p110β suppresses leukaemia *in vitro* and in Pten-deficient mice. (**a**) Colony-forming assays were performed with 1 × 10^4^ (BM) or 5 × 10^4^ (spleen) cells obtained from 7 DPI *Pten*^*Δ/Δ*^ animals. Cells were cultured with added DMSO, BYL719, TGX221,GS1101, NVSPI35, GDC0941 or RAD001 with the following concentrations based on published data: 1, 0.5, 0.1 μM (BYL719, TGX221 and GDC0941); 5, 1, 0.5 μM (GS1101); 0.1, 0.05, 0.01 μM (NVSPI35 and RAD001). Colonies were counted 7 days after incubation. Data are represented as mean±s.e.m. (*n*=3, measured in duplicates) and all data are compared with the DMSO control for statistical analysis. *P<0.05; **P<0.001. Two-way ANOVA test was applied to compare the colony formation. (**b**) Schematic representation of drug treatment. (**c**) Survival of *Pten*^*Δ/Δ*^ animals treated with vehicle (blue; *n*=7; median survival=23 days), BYL719 (green; *n*=8; median survival=32 days), KIN193 (red; *n*=7; median survival=50 days), IC87114 (orange; *n*=7; median survival=28 days) and AS605240 (brown; *n*=6; median survival=23 days). *P<0.05; **P<0.001; ***P<0.0001. The log-rank test was applied to compare survival. (**d**) Spleen weight of vehicle-treated (*n*=4) and KIN193-treated animals (*n*=3) at the moribund stage. *P<0.05. Student’s *t*-test was applied. (**e**) Western blot analysis of Akt signalling in whole BM cells at 7 DPI. Freshly isolated BM cells were treated with DMSO, BYL719, KIN193, GS1101 or NVSPI35 at the 1-μM dose for 2 h (*n*=6) for each treatment. (**f**) Phospho-flow analysis of p-Akt on isoform-selective inhibitor-treated animals. Freshly isolated lineage-negative BM cells were treated with inhibitors as in (**e**) and subjected to flow cytometry for LSK staining and intracellular P-Akt staining (*n*=3) for each, and median fluorescence intensities were normalized to control.

To determine whether our findings using a genetic method could be recapitulated by pharmacologic approaches utilizing PI3K isoform-selective inhibitors at effective doses as published in earlier studies, we first examined the effects of PI3K isoform inhibition on myeloid progenitor function. We cultured BM cells and splenocytes from *Pten*^*Δ/Δ*^ animals in methylcellulose supplemented with myeloid growth factors in the presence of PI3K inhibitors. As reported in previous studies^10^, the pan-PI3K inhibitor GDC0941 and the mTOR inhibitor RAD001 significantly suppressed the increased colony formation arising from Pten deletion in a dose-dependent manner (Fig. 5a). Consistent with our genetic findings, inhibition of p110α with BYL719 (a p110α-selective inhibitor)^32^ and p110δ with GS1101 (ref. 33) had a modest effect on the expansion of myeloid cells in the context of Pten loss (Fig. 5a). Since p110γ is also expressed in leukocytes, we tested the p110γ inhibitor NVSPI35 (ref. 34). Interestingly, inhibition of p110γ with NVSPI35 showed some effect on BM cells, but not on splenocytes (Fig. 5a). Notably, inhibition of p110β with KIN193 (a p110β-selective inhibitor, also known as AZD6482)^14^,^22^ significantly reduced formation of both BM- and spleen-derived myeloid colonies in methylcellulose in a dose-dependent manner (Fig. 5a) suggesting that pharmacologic inhibition of p110β is highly effective in suppressing myeloid cell expansion driven by Pten loss.

To further determine the effects of pharmacologic inhibition of PI3K isoforms *in vivo*, we treated a group of pIpC-induced *Pten*^*Δ/Δ*^ animals with BYL719, KIN193, IC87114 (a p110δ-selective inhibitor in the same class as GS1101, but with better bioavailability in mice)^35^, AS605240 (p110γ-selective inhibitor suitable for *in vivo* studies)^36^ or a vehicle control 10 days after induction (Fig. 5b). All the inhibitors were used at the effective doses *in vivo* as published in earlier studies^36^,^37^,^38^. Treatment with BYL719 (ref. 37), IC87114 (ref. 38) or AS605240 (ref. 36) resulted in a minimal survival benefit compared with vehicle-treated animals (Fig. 5c). Notably, mice treated with KIN193 had a significantly longer survival as compared with mice in any other group (Fig. 5c). Moreover, KIN193-treated animals appeared healthy and had significantly reduced spleen weights and normal-looking spleen architecture compared with vehicle-treated mice (Fig. 5d; Supplementary Fig. 8), consistent with our genetic data that ablation of p110β largely prevented myeloid leukaemia in Pten-deficient mice.

Next, we examined PI3K/Akt signalling in Pten-null BM cells in response to isoform-selective inhibition. As expected, vehicle-treated Pten-null BM cells from *Pten*^*Δ/Δ*^ animals showed markedly increased Akt phosphorylation compared with wild-type BM cells (Fig. 5e). Interestingly, inhibition of either p110α, p110β or p110δ led to a significant reduction of p-Akt, compared with the controls (Fig. 5e). We also examined Akt activation by measuring basal p-Akt levels in LSK cells by intracellular phosopho-flow cytometry after Pten deletion and short-term isoform-selective inhibitor treatment of lineage-negative cells from *Pten*^*Δ/Δ*^ BM. We detected significantly higher basal levels of p-Akt in *Pten*^*Δ/Δ*^ LSK cells compared with WT LSKs, and again inhibition of p110α, p110β, p110δ or p110γ led to a significant reduction of p-Akt compared with the vehicle group (Fig. 5f). Since p110β is not the only isoform responsible for mediating Akt signalling in Pten-deficient BM and HSCs, Akt signalling alone is not sufficient to explain the specific biological effects of p110β ablation or inhibition observed in our study.

### Pten-deficient HSCs depend on the p110β–Rac axis

Recent data suggested that p110α, p110δ and p110γ bind to and are activated by the Ras subfamily of GTPases, while p110β instead binds to and is activated by the Rho subfamily GTPases, Rac1 and CDC42 via its ‘Ras-binding domain’ (RBD)^17^,^21^. Previous studies also reported that an intact RBD was required for signalling and oncogenic transformation by wild-type p110β, suggesting a potential role for the interaction of Rho GTPase with p110β in transformation^39^,^40^. Notably, Rac1 and CDC42 can also be activated downstream of PI3K by PIP3-dependent guanine-nucleotide exchange factors^41^,^42^. It has been previously reported that Rac plays important roles in the homing and survival of HSCs^43^,^44^. Given the significant rescue of HSCs in *Pten*^*Δ/Δ*^*;p110β*^*Δ/Δ*^ mice, we hypothesized that a unique positive-feedback signalling loop might exist between p110β and Rac, in which p110β is activated by Rac and Rac could in turn be activated by the phosphoinoside products of p110β in the setting of Pten-null haematopoietic cells.

**Figure 6:**
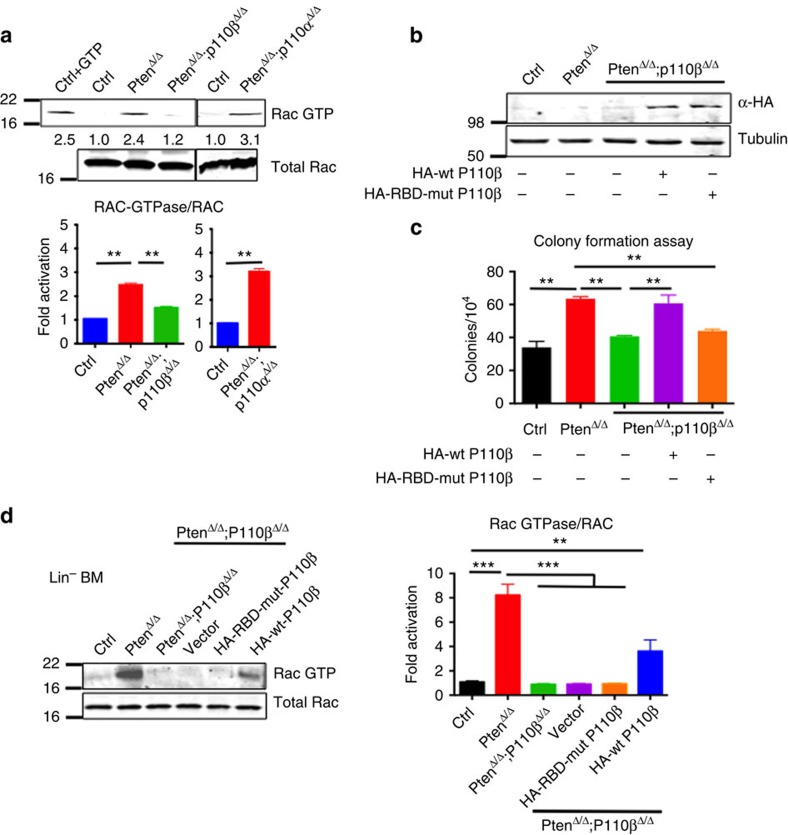
Pten-deficient haematopoietic cells depend on the p110β–Rac1 signalling axis. (**a**) Rac–GTP activity assay was performed on BM after 7 DPI in the corresponding genotypes. Rac–GTP and total Rac were measured and normalized (*n*=3; mean with s.e.m.). Two-way ANOVA test was applied to compare the RAC-GTPase/RAC levels. (**b**) *Pten*^*Δ/Δ*^*;p110β*^*Δ/Δ*^ BM cells were transduced with retroviruses expressing HA-tagged wild-type P110β or RBD-mutant P110β, and briefly selected in the presence of puromycin. HA immunoblotting was performed to show expression of RBD-mutant and wild-type P110β (*n*=3 for each group). (**c**) Cells from WT control, *Pten*^*Δ/Δ*^ or *Pten*^*Δ/Δ*^*;p110β*^*Δ/Δ*^ animals were cultured in methylcellulose with myeloid growth factors in the presence of puromycin for 7 days, and colonies were counted at the end of day 7 (*n*=3 for each group). Two-way ANOVA test was applied to compare the colony numbers. (**d**) Lineage-negative cells were isolated from WT control, *Pten*^*Δ/Δ*^ or *Pten*^*Δ/Δ*^*;p110β*^*Δ/Δ*^ mice, and the Rac–GTP activity assay was performed as in (**a**). Rac–GTP and total Rac were measured and normalized to WT (*n*=6; mean with s.e.m.). Two-way ANOVA test was applied to compare the RAC-GTPase/RAC levels. **P< 0.01, ***P<0.001

Notably, we detected higher levels of Rac–GTP in the BM of *Pten*^*Δ/Δ*^ mice, which could be suppressed by deletion of p110β but not p110α (Fig. 6a). To investigate whether the binding of p110β to Rac is important in mediating p110β activity in Pten-deleted BM cells, we mutated the two highly conserved key residues within the p110β RBD to generate a p110β–S205D/K224A double mutant lacking the binding activity to Rac1 (ref. 21), and performed an add-back experiment with either wild-type or RBD-mutant p110β in BM cells derived from *Pten*^*Δ/Δ*^*;p110β*^*Δ/Δ*^ mice (Fig. 6b; Supplementary Fig. 10). Colony-forming assays revealed that, while adding back a wild-type p110β in *Pten*^*Δ/Δ*^*;p110β*^*Δ/Δ*^ BM cells restored myeloid colony numbers comparable to those of *Pten*^*Δ/Δ*^ BM cells, the RBD-mutant p110β failed to rescue colony formation (Fig. 6c). To determine whether p110β affects Rac–GTP levels in HSCs/progenitor cells (HSPCs), we performed the Rac–GTP assay either on Lin-negative *Pten*^*Δ/Δ*^ BM cells or *Pten*^*Δ/Δ*^*;p110β*^*Δ/Δ*^ cells expressing wild-type p110β and RBD-mutant p110β. We detected higher levels of Rac–GTP in the Pten-deficient cells compared with WT control cells, and these levels were significantly reduced in Pten^Δ/Δ^;p110β^Δ/Δ^ cells. Adding back wild-type p110β to *Pten*^*Δ/Δ*^*;p110β*^*Δ/Δ*^ HSC/P cells, partially rescued Rac–GTP levels but adding back RBD-mutant p110β failed to rescue Rac activity (Fig. 6d; Supplementary Fig. 10) Together, these data suggest that the interaction of p110β with Rac plays an important role in mediating the myeloid clonogenic activity driven by Pten loss.

**Figure 7:**
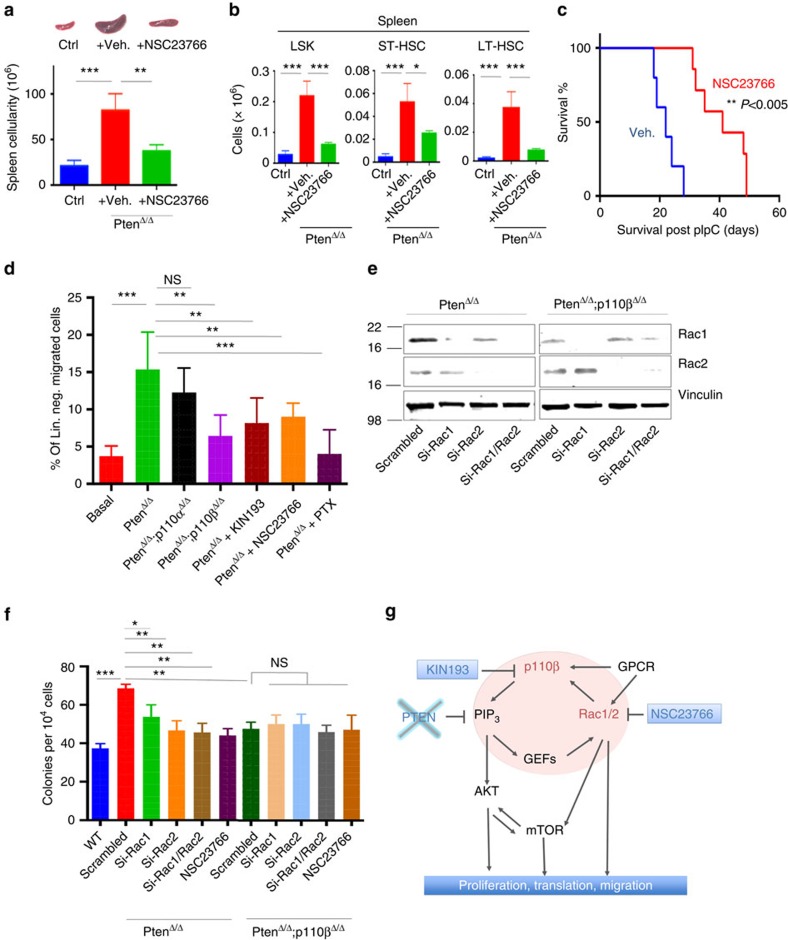
Pten-deficient haematopoietic cells depend on the p110β–Rac1 signalling axis. (**a**) NSC23766 was given at 2.5 μg g^−1^ daily intraperitoneally (IP). Spleen size and cellularity of vehicle-treated (*n*=3) and NSC23766-treated *Pten*^*Δ/Δ*^ animals (*n*=3) at 17 DPI. *P<0.05; **P<0.001; ***P<0.0001. Two-way ANOVA test was applied to compare spleen size. (**b**) Quantification of the numbers of LSK (Lin^–^Sca-1^+^c-kit^+^), ST-HSC (CD150^–^CD48^−^Lin^−^Sca-1^+^c-kit^+^) or LT-HSC (CD150^+^CD48^−^Lin^−^Sca-1^+^c-kit^+^) cells in the spleens of control, *Pten*^*Δ/Δ*^ animals treated with vehicle, or *Pten*^*Δ/Δ*^ animals treated with NSC23766 at 17 DPI, determined by flow cytometry. The numbers represent total cell counts per spleen (*n*=3; ctrl, *Pten*^*Δ/Δ*^ vehicle and NSC23766 treated). (**c**) Survival of *Pten*^*Δ/Δ*^ animals treated with vehicle (blue; *n*=7; median survival=22 days) or NSC23766 (red; *n*=7; median survival=42 days). **P<0.005. The log-rank test was applied to compare survival. (**d**) Lineage-negative BM cells were isolated from control, *Pten*^*Δ/Δ*^ or *Pten*^*Δ/Δ*^*:P110β*^*Δ/Δ*^ *Pten*^*Δ/Δ*^*:P110α*^*Δ/Δ*^ on 7 DPI, and lineage-negative cells were pretreated for 3 h with KIN193, NSC23766 (50 μM) or pertussis toxin (PTX; 100 ng ml^−1^). Migration towards the lower chamber (supplemented with CXCL12, 50 nM) was measured as the fraction of migrated cells relative to untreated Pten-null bone marrow-derived cells. Numbers of replicates are *n*=4 (basal, *Pten*^*Δ/Δ*^ no Cxcl12; *Pten*^*Δ/Δ*^+PTX), 10 (*Pten*^*Δ/Δ*^), 6 (*Pten*^*Δ/Δ*^*:P110β*^*Δ/Δ*^) and 6 (*Pten*^*Δ/Δ*^+KIN193). Statistical comparisons were all performed relative to the *Pten*^*Δ/Δ*^ group. (**e**) *Pten*^*Δ/Δ*^ and *Pten*^*Δ/Δ*^*;p110β*^*Δ/Δ*^ BM cells were transfected with two different siRNAs targeting scrambled, mouse Rac1, mouse Rac2 alone, or both Rac1 and Rac2 and subjected to immunoblotting 48 h later. (**f**) *Pten*^*Δ/Δ*^ and *Pten*^*Δ/Δ*^*;p110β*^*Δ/Δ*^ BM cells from knockdown were transfected with two different siRNAs targeting scrambled, mouse Rac1, mouse Rac2 alone, or both Rac1 and Rac2, or treated with NSC23766 (50 μM) and then colony assays were performed in methylcellulose with myeloid growth factors. Colonies were counted after 7 days (*n*=3) for each group. Two-way ANOVA test was applied to calculate colony formation. (**g**) A working model of signalling cascades downstream of P110β and the signalling loop of P110β and Rac1.

To further investigate the functional dependency of Pten-deleted leukaemic cells on the p110β–Rac axis, we utilized NSC23766, a potent Rac inhibitor^45^ in our Pten-null model (Fig. 7a). We found that treatment of these mice for 10 days led to a reduced disease burden, as demonstrated by reduced spleen size and cellularity (Fig. 7a; Supplementary Fig. 9). Similarly, treatment of *Pten*^*Δ/Δ*^ mice with NSC23766 resulted in a significant reduction of HSC and myeloid progenitor numbers in the spleen compared with vehicle controls (Fig. 7b; Supplementary Fig. 9). Treatment with NSC23766 also led to a significantly prolonged survival of *Pten*^*Δ/Δ*^ mice (Fig. 7c), recapitulating the findings for genetic ablation or pharmacological inhibition of p110β in Pten-null animals.

Since Rac is required for p110β activation downstream of GPCRs^21^, we assessed the functional importance of the Rac–p110β signalling axis in HSPC function in response to activation of CXCR4, a GPCR important in the regulation of HSPCs. We used Transwell migration assays with CXCL12, a potent chemo-attractant of stem cells that signals through CXCR4. Lineage-negative BM cells from Pten^Δ/Δ^ mice showed increased migration towards CXCL12 compared with WT control cells (Fig. 7d). This migration was abolished by the GPCR inhibitor pertussis toxin (PTX; Fig. 7d). Interestingly, we observed significantly reduced migration of Pten^Δ/Δ^;p110β^Δ/Δ^ cells, and of *Pten*^*Δ/Δ*^ cells treated with either KIN193 or NSC23766, compared with Pten^Δ/Δ^ and *Pten*^*Δ/Δ*^*;p110α*^*Δ/Δ*^ cells (Fig. 7d). This suggests that deletion of p110β, or pharmacologic inhibition of either p110β or Rac, partially interferes with the migration of *Pten*^*Δ/Δ*^ cells towards a CXCL12 gradient, likely through perturbed GPCR signalling.

Because murine models of haematopoietic-specific Rac1 and Rac2 deficiency have revealed differential roles of Rac proteins in terms of HSPC function, we wanted to understand which Rac isoform is more important in the absence of Pten. To this end, we used siRNA to knockdown either Rac1 or Rac2, or both, and performed colony assays on *Pten*^*Δ/Δ*^ and *Pten*^*Δ/Δ*^*;p110β*^*Δ/Δ*^ BM cells (Fig. 7e,f; Supplementary Fig. 10). We also tested the Rac inhibitor NSC23766, which targets both Rac1 and Rac2 (ref. 46). Knockdown of either Rac1 or Rac2, or their combined knockdown or pharmacological inhibition significantly reduced colony formation by Pten^Δ/Δ^ cells to levels obtained with *Pten*^*Δ/Δ*^*;p110β*^*Δ/Δ*^ BM cells (Fig. 7f). However, knockdown of Rac1 or Rac2, the combination, or NSC23766 treatment did not further suppress colony formation beyond the effects of p110β deletion (Fig. 7f), suggesting that there is no additive or synergistic effect of Rac inhibition with p110β deficiency in *Pten*^*Δ/Δ*^ cells. Together, these results suggest that p110β–Rac1/2 work in concert to mediate the effects of Pten loss in promoting myeloid neoplasia.

## Discussion

We and others have reported that Pten-deficient solid tumours frequently rely on p110β (refs 20, 23, 47). In this study, we report for the first time an essential role for p110β in promoting haematologic neoplasia driven by Pten deletion in HSCs despite the expression of four different PI3K isoforms in haematopoietic cells. We have also found that p110β contributes to HSC depletion in the BM after Pten deletion. Interestingly, we found that Mx1–Cre-mediated deletion of p110β in HSPCs of animals that are WT for Pten does not significantly affect blood counts. In fact, these animals appear healthy for many months after excision, suggesting that targeting p110β may lead to an effective therapy for myeloid leukaemia with little toxicity to normal HSCs.

Despite the marked impact of genetic deletion or pharmacologic inhibition of p110β on MPN, and the significantly delayed onset of leukaemia, a fraction of *Pten*^*Δ/Δ*^*;p110β*^*Δ/Δ*^ animals succumbed to T-ALL at a later time. Berenjeno *et al*.^48^ showed that in Pten^+/–^ animals, inactivation of p110β led to reduced PIP3 generation in lymphoma tissues, but had little impact on lymphoma formation. It is possible that these tumours become p110β independent through the acquisition of secondary alterations. In fact, Yilmaz *et al*.^10^ have documented the presence of cytogenetic alterations in leukaemic blasts from *Pten*^*Δ/Δ*^ animals. Alternatively, isoform dependency may shift with cell differentiation. For example, the isoform dependency in the skin hamartoma driven by Pten loss changed from a p110β dependency in the basal layer of the epidermis to a p110α dependency in the suprabasal cells as the basal cells underwent stepwise differentiation to become suprabasal cells^37^. In this study, we also provide evidence that neither p110α nor p110β has any effect on T-ALL driven by T lymphocyte-specific deletion of Pten using Lck–Cre. In this system, it has been shown that p110δ and p110γ contribute to T-ALL induced by Pten loss in T cells^28^. These studies provide additional data that accentuate the distinct roles of p110β in the HSCs and in myeloid and lymphoid tumour initiation in the absence of Pten.

Interestingly, we found that inhibition of p110β, p110α or p110δ could similarly reduce p-Akt in Pten-deficient BM and HSCs, suggesting an Akt-independent pathway specific to p110β is important in Pten-deficient HSCs in promoting myeloid neoplasia. We report a new mechanistic insight that may explain the unique role of p110β in this setting. Since p110β binds to Rac rather than to Ras via its RBD, unlike the other class I PI3K isoforms^21^, we investigated the role of the p110β–Rac axis in the setting of myeloid neoplasia induced by Pten loss in HSCs. Notably, Rac signalling is not only important for the activation of p110β but it itself is also activated by PIP3 via PIP3-activated guanine-nucleotide exchange factors, forming a potential signalling loop (Fig. 7g)^14^,^49^. We found strong evidence that this loop is indeed active in our Pten-deficient model. We show that Rac was activated in Pten-null BM cells, and this activation was suppressed in Pten/p110β double KOs, but not in Pten/p110α double KOs. This hypothesis was further supported by our finding that only wild-type p110β, but not the RBD mutant of p110β, rescued colony formation in Pten/p110β-deficient BM cells. Notably, the effect of the RBD-mutant p110β on inhibiting colony formation in Pten-null BM cells is comparable to that of p110β deletion. An intact RBD was reported to be required for membrane localization of p110β for both signalling and oncogenic transformation by wild-type p110β in cultured cells^40^. Our data suggest that the interaction of p110β–Rac may play an important role in mediating p110β activity downstream of GPCRs and tyrosine kinases in the context of Pten deficiency. Moreover, pharmacologic inhibition of p110β or Rac in Pten-deficient mice resulted in a strikingly similar functional rescue *in vivo*, with a reduction in extramedullary haematopoiesis in the spleen and improved survival.

Of the three isoforms of Rac family GTPases, Rac2 is expressed specifically in haematopoietic cells, while Rac1 and Rac3 are ubiquitously expressed^44^,^50^. The Rac inhibitor NSC23766 targets all three Rac members: Rac1, 2 and 3 (ref. 46). Both Rac1 and CDC42 have been shown to bind p110β in a recent study^21^. Binding to Rac2/3 was not tested, but might also be expected, based on the homology of their effector domains with that of Rac1. Previous studies have suggested that Rac1 and Rac2 play both distinct and overlapping roles in HSCs and progenitor cells, while the role of Rac3 in haematopoiesis has not been defined^44^,^51^. It also has been shown that targeting both Rac1 and Rac2 was effective in a mouse model of BCR–ABL-induced MPN, as well as in a mouse model of MLL–AF9 AML^46^,^52^. Interestingly, our data show that the effect of knockdown of Rac2 is comparable to that of combined knockdown of both Rac1 and Rac2, or a pan-Rac inhibitor NSC23766. Since Rac2 is primarily expressed in haematopoietic cells, our data suggest that Rac2 could potentially be a better pharmacologic target with reduced toxicity.

Yilmaz *et al*. have shown that the mTOR inhibitor rapamycin can rescue HSC depletion and can suppress the development of leukaemia *in vivo*. More recently, the Armstrong and Morrison groups reported that mTORC1 and mTORC2 play critical roles in haematopoiesis and Pten-loss-driven leukaemogenesis, respectively^53^,^54^. By ablation or inhibition of p110β, we obtained similar results suggesting that the activation of mTOR in HSCs by Pten loss may be mediated largely by p110β. A recent study demonstrated that Rac1 regulates the activity of both mTORC1 and mTORC2 (ref. 55), providing a potential Akt-independent link between p110β–Rac and mTOR. Together, these data suggest that p110β–Rac acts upstream of Akt and mTOR. We feel that our data are most consistent with the working model shown in Fig. 7g, in which p110β works in a signalling loop with Rac to generate the key signals arising from Pten loss. Notably, these signals include both Akt-dependent and Akt-independent pathways leading to cell proliferation and migration.

In summary, our results provide the first evidence that PI3K–p110β plays an essential role in controlling HSC function in the setting of Pten loss^56^. We have also uncovered a specific role for p110β in myeloid leukaemia induced by Pten deficiency. In contrast, we found that p110β is dispensable for T-ALL induced by Pten loss. Most importantly, our data show that a p110β–Rac signalling loop is important for the induction of myeloid neoplasia in the absence of Pten. Thus, second-generation p110β- or Rac-selective inhibitors may interrupt this loop, thereby providing a promising new therapeutic strategy for Pten-deficient myeloid leukaemias while preserving normal haematopoiesis.

## Methods

### Animal experiments

Mice were housed in a pathogen-free animal facility at Dana-Farber Cancer Institute (DFCI). All animal experiments conformed to the relevant regulatory standards, guidelines and regulations, and were approved by the DFCI-Institutional Animal Care and Use Committee (IACUC). Dead and moribund animals were included in survival curves. Pten^fl/fl^ mice (obtained from Hong Wu’s Laboratory) were crossed with Pten^fl/+^ animals carrying an Mx-1-Cre transgene (obtained from Dr Gary Gilliland’s laboratory) to obtain Mx-1-Cre^+^;Pten^fl/fl^ mice (*Pten*^*Δ/Δ*^). p110α and p110β mutants were obtained by crossing Mx-1-Cre^+^;Pten^fl/+^ mice with p110α^fl/fl^, p110β^fl/fl^ animals to obtain Mx-1-Cre^+^;Pten^fl/fl^;p110α^fl/fl^ (*Pten*^*Δ/Δ*^*;p110α*^*Δ/Δ*^) and Mx-1-Cre^+^;Pten^fl/fl^;p110β^fl/fl^ animals (*Pten*^*Δ/Δ*^*;p110β*^*Δ/Δ*^). P110δ germline knockout animals (James Ihle’s laboratory) were backcrossed to the C57BL/6 background for more than nine generations and then crossed with Mx-1-Cre^+^;Pten^fl/+^ to obtain Mx-1-Cre^+^;Pten^fl/fl^;p110δ^−/−^ animals (*Pten*^*Δ/Δ*^*;p110δ*^*−/−*^). Thirty day-old male and female animals were induced by intraperitoneal injection with pIpC (25 mg g^−1^; GE Healthsciences) every other day for 5 days. WT;Mx-1-Cre^+^ mice were used as controls for all experiments. Peripheral blood was obtained by retro-orbital venous blood sampling. Peripheral blood counts were analysed on a Hemavet 950FS blood analyzer (Drew Scientific).

### Flow cytometry

All data acquisition was performed on a LSRII (BD) flow cytometer, and results were analysed using FlowJo v.8.8.7 (Tree Star). Antibodies used for flow cytometry were directly coupled and directed against B220 (APC, BD Pharmingen), cKit (PE-Cy7, BioLegend), CD3 (PE-Cy7, BD Bioscience), CD4 (APC-H7, BD Pharmingen), CD8 (ECD, Beckman Coulter), CD11b (PE, BD Bioscience), CD16/32 (PE, eBioscience), CD34 (FITC, BD Pharmingen), CD45.1 (FITC, BD Pharmingen), CD45.2 (PerCP-Cy5.5, BD Pharmingen), CD48 (APC-Cy7, BD Pharmingen), CD127 (ECD, BD Pharmingen), CD150 (PerCP-Cy5.5, BioLegend), Gr1 (APC-Alexa 700, BD Bioscience), lineage cocktail (APC, BD Pharmingen), Sca1 (Brilliant Violet 421, BioLegend), Ki67 (Alexa 488, BD Pharmingen). Dead cells were excluded using either DAPI or Vivid-Aqua (Invitrogen) staining.

### Chemotaxis assays

Fresh BM cells were isolated from mutant mice and from control animals, and lineage-positive cells were depleted using magnetic separation (lineage depletion kit, Miltenyi Biotec) and were resuspended in PBS containing 0.5% BSA. Lineage-negative cells (0.5–1 million) were pretreated for 3 h at 37 °C with the p110β-specific inhibitor KIN193 (1 μM), NSC23766 (50 μM) PTX from Bordetella pertussis (100 ng ml^−1^) or dimethylsulphoxide (DMSO; 1:1,000), or directly loaded (10^5^ cells in 100 μl) in the top chamber of a 6.5-mm diameter 8-μm pore polycarbonate Transwell insert (Corning Costar); inserts with cells were then placed in wells containing CXCL12 (50 mM, PeproTech), and incubated for 3 h at 37 °C. Cells migrating from the top to the bottom chamber were collected and quantified using a Countess cell counting machine (Invitrogen).

### Compounds

TGX221 was obtained from Chemdea; NSC23766 and IC87114 from Selleck Chemicals; GDC0941 from MedChemexpress; KIN193 from Haoyuan Chemexpress; NVP-BYL719, NVSPI35, AS605240 and IC87114 were synthesized by Chemitek (Indianapolis, USA). Mice were dosed once by intraperitoneal injection of KIN193 (20 mg kg^−1^) formulated in 7.5% NMP (Sigma-Aldrich), 40% polyethylene glycol 400 (Sigma-Aldrich) and 52.5% dH_2_O; once by intraperitoneal injection of NSC23766 (2.5 mg kg^−1^), or gavaged once daily with NVP-BYL719 (45 mg kg^−1^) in 0.5% methylcellulose; AS605240 (50 mg kg^−1^) in 0.5% carboxymethylcellulose/0.25% Tween-20; or IC87114 (45 mg kg^−1^) dissolved in 75% polyethylene glycol.

### Colony-forming assays

BM and spleen cells were collected, subjected to red-cell lysis and resuspended in Iscove’s modified Dulbecco’s medium/10% fetal bovine serum/5% penicillin–streptomycin. Cells were plated in the presence of inhibitors in duplicate in M3434 methylcellulose media (Stemcell Technologies) at 1 × 10^4^ cells per dish for BM and 5 × 10^4^ cells per dish for spleen cells. Colonies were scored after 7 days.

### Rac activation assay

BM cells from corresponding mice at 7 DPI of pIpC were collected and immediately subjected to Rac1 activation assay with the Rac1 activation assay kit (Millipore) according to the manufacturer’s instructions.

### Western blotting

Whole-cell protein lysates were prepared from single-cell suspensions of BM and splenocytes from corresponding mice, and from age-matched WT C57 Bl/6 mice. Western blotting was performed with the slight changes as follows: 0.45-μM nitrocellulose membranes (Millipore) were used to transfer the proteins, this followed by blocking 1 h at room temperature in 1 × TBS, 5% nonfat milk. Primary antibodies used were as follows: p110α (1:1,000, catalogue no. 4249; Cell Signaling), p110β (1:1,000, catalogue no. sc-602; Santa Cruz), p110δ (1:1,000, catalogue no. sc-55589; Santa Cruz), Akt (1:1,000, catalogue no. 9272; Cell Signaling), phospho-Akt (Ser473; 1:500, catalogue no. 4050; Cell Signaling), S6 (1:1,000, catalogue no. 2217; Cell Signaling), phospho-S6 (catalogue no. 2211; Cell Signaling), Rac1 (1:500, catalogue no. 05-389; Millipore), Rac2 (1:500, catalogue no. sc-96; Santa Cruz), α-HA (1:2,000 catalogue no. H6908; Sigma-Aldrich), α-tubulin (1:5,000 catalogue no. T9026; Sigma-Aldrich) and vinculin (1:5,000 catalogue no. sc-73614, Santa Cruz). The directly fluorophore-conjugated secondary antibodies: anti-mouse 680 and anti-rabbit 800 (LICOR) were used at dilutions of 1:5,000. LICOR Odyssey instrument was used to develop the membranes. Signal intensity with background correction was quantified using LICOR Image Studio software. Whole Western Blot pictures are shown in Supplementary Fig. 10.

### SiRNA knockdown

Two separate SiRNAs, targeting mouse Rac1 (J-041170-05-0002 and J-041170-06-0002) and Rac2 (J-041171-09-0002 and J-041171-10-0002) were ordered from Dharmacon (Dharmacon, GE Healthcare) transfected in Invitrogen X15 media to freshly isolated whole bone marrow (WBM) cells using RNAiMAX (Invitrogen) in 10-cm plates according to the manufacturer’s instructions. Forty-eight hours later after transfection, cells were subjected to immunoblotting using Rac1 or Rac2 antibodies or colony-forming experiments.

### Histology

Recipient mice were killed at the indicated time points, or when they began to show signs of disease. Organs were fixed in formalin, and histology slides were prepared and stained at the Brigham and Women’s Rodent Histology Core Facility. Digital images were acquired on a Nikon Eclipse E400 microscope equipped with a digital camera and analysed using Spot Advanced software.

### Immunohistochemistry

For histological analyses, formalin-fixed tissue sections were embedded in paraffin, sectioned and stained with haematoxylin and eosin by the Dana-Farber/Harvard Cancer Center Rodent Histopathology Core.

### P-Akt flow cytometry analysis of LSK cells

Phospho-flow cytometry was performed as previously described^56^, with the following modifications: lineage-negative BM cells from mutant animals were isolated using lineage depletion kit (Miltenyi Biotec) and serum starved for 1 h, and treated for 2 h with 1 μM of BYL719, KIN193, GS1101 and NVSPI3. Cells were then fixed with 4% paraformaldehyde, and permeabilized with cold 100% acetone. Cells were than stained simultaneously with c-kit, Sca-1 and anti-mouse P-Akt (Alexa 647; 1:100 dilution, cat. no. 2337, Cell Signaling). All data acquisition was performed on a LSRII (BD) flow cytometer, and results were analysed and basal level of P-Akt was calculated as normalized to WT cells by calculating median fluorescent intensity using FlowJo v.8.8.7 (Tree Star).

### Long-term competitive repopulation assays

Recipient mice (4-6 weeks old female mice; B6.SJL strain) received two doses of 540 rads each, delivered 3 h apart. Nucleated BM cells from control and *Pten*^*Δ/Δ*^ mice (C57 Bl/6) or from compound mutant mice (*Pten*^*Δ/Δ*^*; p110α*^*Δ/Δ*^, *Pten*^*Δ/Δ*^*;p110β*^*Δ/Δ*^ and *Pten*^*Δ/Δ*^*;p110δ*^*−/−*^) were mixed with wild-type competitor BM-nucleated cells (B6.SJL), and were injected into the retro-orbital venous sinus of irradiated recipients. Peripheral blood was obtained retro-orbitally every 4 weeks, subjected to red-cell lysis and analysed by flow cytometry to assess donor cell engraftment for up to 20 weeks after transplantation. Pilot experiments showed that Pten-mutant animals had a greatly reduced repopulation capacity; therefore, an excess of mutant cells over control cells was used. Each recipient mouse received 1 × 10^6^ Pten mutant, ctrl or compound mutant BM-nucleated cells plus 2 × 10^5^ competitor cells. Tissues from recipient mice were collected and stained for pathological examination.

### Plasmid constructs

Mutations were generated in the pBabe–HA wild-type P110β by site-directed mutagenesis to obtain p110β–S205D/K224A double mutant to obtain Ras-binding mutant P110β. This plasmid was used to transduce *Pten*^*Δ/Δ*^*;p110β*^*Δ/Δ*^ BM cells and for colony-forming experiments.

### Statistical methods

Two-tailed *T*-tests (two groups) and analysis of variance (ANOVA) tests (multiple groups) and log-rank statistics (Mantel-Cox test; survival studies) were used, and calculated with the use of Prism 6.0 (GraphPad). Data are represented as mean±s.d. unless otherwise noted. *P<0.05; **P<0.01; ***P<0.001.

## Additional information

**How to cite this article:** Yuzugullu, H. *et al*. A PI3K p110β–Rac signalling loop mediates Pten-loss-induced perturbation of haematopoiesis and leukaemogenesis. *Nat. Commun.* 6:8501 doi: 10.1038/ncomms9501 (2015).

## Supplementary Material

Supplementary Information Supplementary Figures 1-10

## References

[b1] Martelli A. M. . Phosphoinositide 3-kinase/Akt signaling pathway and its therapeutical implications for human acute myeloid leukemia. Leukemia 20, 911–928 (2006) . 16642045 10.1038/sj.leu.2404245

[b2] Peng C. . PTEN is a tumor suppressor in CML stem cells and BCR-ABL-induced leukemias in mice. Blood 115, 626–635 (2010) . 19965668 10.1182/blood-2009-06-228130 PMC2810991

[b3] Gutierrez A. . High frequency of PTEN, PI3K, and AKT abnormalities in T-cell acute lymphoblastic leukemia. Blood 114, 647–650 (2009) . 19458356 10.1182/blood-2009-02-206722 PMC2713461

[b4] Dahia P. L. . PTEN is inversely correlated with the cell survival factor Akt/PKB and is inactivated via multiple mechanismsin haematological malignancies. Hum. Mol. Genet. 8, 185–193 (1999) . 9931326 10.1093/hmg/8.2.185

[b5] Patel J. P. . Prognostic relevance of integrated genetic profiling in acute myeloid leukemia. N. Engl. J. Med. 366, 1079–1089 (2012) . 22417203 10.1056/NEJMoa1112304 PMC3545649

[b6] Li Y. . Epigenetic silencing of microRNA-193a contributes to leukemogenesis in t(8;21) acute myeloid leukemia by activating the PTEN/PI3K signal pathway. Blood 121, 499–509 (2013) . 23223432 10.1182/blood-2012-07-444729

[b7] Noguera N. I. . Nucleophosmin/B26 regulates PTEN through interaction with HAUSP in acute myeloid leukemia. Leukemia 27, 1037–1043 (2013) . 23183427 10.1038/leu.2012.314

[b8] Yang J. . A reappraisal by quantitative flow cytometry analysis of PTEN expression in acute leukemia. Leukemia 21, 2072–2074 (2007) . 17611573 10.1038/sj.leu.2404740

[b9] Xu Q., Simpson S. E., Scialla T. J., Bagg A. & Carroll M. Survival of acute myeloid leukemia cells requires PI3 kinase activation. Blood 102, 972–980 (2003) . 12702506 10.1182/blood-2002-11-3429

[b10] Yilmaz O. H. . Pten dependence distinguishes haematopoietic stem cells from leukaemia-initiating cells. Nature 441, 475–482 (2006) . 16598206 10.1038/nature04703

[b11] Zhang J. . PTEN maintains haematopoietic stem cells and acts in lineage choice and leukaemia prevention. Nature 441, 518–522 (2006) . 16633340 10.1038/nature04747

[b12] Tang P. . Differential roles of Kras and Pten in murine leukemogenesis. Leukemia 27, 1210–1214 (2013) . 23183424 10.1038/leu.2012.316 PMC3650112

[b13] Vardiman J. W. . The 2008 revision of the World Health Organization (WHO) classification of myeloid neoplasms and acute leukemia: rationale and important changes. Blood 114, 937–951 (2009) . 19357394 10.1182/blood-2009-03-209262

[b14] Thorpe L. M., Yuzugullu H. & Zhao J. J. PI3K in cancer: divergent roles of isoforms, modes of activation and therapeutic targeting. Nat. Rev. Cancer 15, 7–24 (2014) . 10.1038/nrc3860 PMC4384662 25533673

[b15] Utermark T. . The p110alpha and p110beta isoforms of PI3K play divergent roles in mammary gland development and tumorigenesis. Genes Dev. 26, 1573–1586 (2012) . 22802530 10.1101/gad.191973.112 PMC3404385

[b16] Castellano E. . Requirement for interaction of PI3-kinase p110alpha with RAS in lung tumor maintenance. Cancer Cell 24, 617–630 (2013) . 24229709 10.1016/j.ccr.2013.09.012 PMC3826036

[b17] Gritsman K. . Hematopoiesis and RAS-driven myeloid leukemia differentially require PI3K isoform p110alpha. J. Clin. Invest. 124, 1794–1809 (2014) . 24569456 10.1172/JCI69927 PMC3973083

[b18] Ciraolo E. . Phosphoinositide 3-kinase p110beta activity: key role in metabolism and mammary gland cancer but not development. Sci. Signal. 1, ra3 (2008) . 18780892 10.1126/scisignal.1161577 PMC2694958

[b19] Guillermet-Guibert J. . The p110beta isoform of phosphoinositide 3-kinase signals downstream of G protein-coupled receptors and is functionally redundant with p110gamma. Proc. Natl Acad. Sci. USA 105, 8292–8297 (2008) . 18544649 10.1073/pnas.0707761105 PMC2448830

[b20] Jia S. . Essential roles of PI(3)K-p110beta in cell growth, metabolism and tumorigenesis. Nature 454, 776–779 (2008) . 18594509 10.1038/nature07091 PMC2750091

[b21] Fritsch R. . RAS and RHO families of GTPases directly regulate distinct phosphoinositide 3-kinase isoforms. Cell 153, 1050–1063 (2013) . 23706742 10.1016/j.cell.2013.04.031 PMC3690480

[b22] Ni J. . Functional characterization of an isoform-selective inhibitor of PI3K-p110beta as a potential anticancer agent. Cancer Discov. 2, 425–433 (2012) . 22588880 10.1158/2159-8290.CD-12-0003 PMC3384541

[b23] Wee S. . PTEN-deficient cancers depend on PIK3CB. Proc. Natl Acad. Sci. USA 105, 13057–13062 (2008) . 18755892 10.1073/pnas.0802655105 PMC2529105

[b24] Furman R. R. . Idelalisib and rituximab in relapsed chronic lymphocytic leukemia. N. Engl. J. Med. 370, 997–1007 (2014) . 24450857 10.1056/NEJMoa1315226 PMC4161365

[b25] Zhao J. J. . The p110alpha isoform of PI3K is essential for proper growth factor signaling and oncogenic transformation. Proc. Natl Acad. Sci. USA 103, 16296–16300 (2006) . 17060635 10.1073/pnas.0607899103 PMC1637576

[b26] Jou S. T. . Essential, nonredundant role for the phosphoinositide 3-kinase p110delta in signaling by the B-cell receptor complex. Mol. Cell. Biol. 22, 8580–8591 (2002) . 12446777 10.1128/MCB.22.24.8580-8591.2002 PMC139888

[b27] Kuhn R., Schwenk F., Aguet M. & Rajewsky K. Inducible gene targeting in mice. Science 269, 1427–1429 (1995) . 7660125 10.1126/science.7660125

[b28] Subramaniam P. S. . Targeting nonclassical oncogenes for therapy in T-ALL. Cancer Cell 21, 459–472 (2012) . 22516257 10.1016/j.ccr.2012.02.029

[b29] Hagenbeek T. J. & Spits H. T-cell lymphomas in T-cell-specific Pten-deficient mice originate in the thymus. Leukemia 22, 608–619 (2008) . 18046443 10.1038/sj.leu.2405056

[b30] Hagenbeek T. J. . The loss of PTEN allows TCR alphabeta lineage thymocytes to bypass IL-7 and Pre-TCR-mediated signaling. J. Exp. Med. 200, 883–894 (2004) . 15452180 10.1084/jem.20040495 PMC2213281

[b31] Tesio M. . Pten loss in the bone marrow leads to G-CSF-mediated HSC mobilization. J. Exp. Med. 210, 2337–2349 (2013) . 24127490 10.1084/jem.20122768 PMC3804947

[b32] Juric D. . Phase I study of BYL719, an alpha-specific PI3K inhibitor, in patients with PIK3CA mutant advanced solid tumors: preliminary efficacy and safety in patients with PIK3CA mutant ER-positive (ER+) metastatic breast cancer (MBC). Cancer Res. 72, P6-10-07 (2012) .

[b33] Lannutti B. J. . CAL-101, a p110delta selective phosphatidylinositol-3-kinase inhibitor for the treatment of B-cell malignancies, inhibits PI3K signaling and cellular viability. Blood 117, 591–594 (2011) . 20959606 10.1182/blood-2010-03-275305 PMC3694505

[b34] Bruce I. . Development of isoform selective PI3-kinase inhibitors as pharmacological tools for elucidating the PI3K pathway. Bioorg. Med. Chem. Lett. 22, 5445–5450 (2012) . 22863202 10.1016/j.bmcl.2012.07.042

[b35] Sujobert P. . Essential role for the p110delta isoform in phosphoinositide 3-kinase activation and cell proliferation in acute myeloid leukemia. Blood 106, 1063–1066 (2005) . 15840695 10.1182/blood-2004-08-3225

[b36] Camps M. . Blockade of PI3Kgamma suppresses joint inflammation and damage in mouse models of rheumatoid arthritis. Nat. Med. 11, 936–943 (2005) . 16127437 10.1038/nm1284

[b37] Wang Q. . Spatially distinct roles of class Ia PI3K isoforms in the development and maintenance of PTEN hamartoma tumor syndrome. Genes Dev. 27, 1568–1580 (2013) . 23873941 10.1101/gad.216069.113 PMC3731546

[b38] Ali K. . Essential role for the p110delta phosphoinositide 3-kinase in the allergic response. Nature 431, 1007–1011 (2004) . 15496927 10.1038/nature02991

[b39] Kang J. H. . Phosphorylation of Rho-associated kinase (Rho-kinase/ROCK/ROK) substrates by protein kinases A and C. Biochimie 89, 39–47 (2007) . 16996192 10.1016/j.biochi.2006.08.003

[b40] Denley A., Kang S., Karst U. & Vogt P. K. Oncogenic signaling of class I PI3K isoforms. Oncogene 27, 2561–2574 (2008) . 17998941 10.1038/sj.onc.1210918

[b41] Klarlund J. K. . Signaling by phosphoinositide-3,4,5-trisphosphate through proteins containing pleckstrin and Sec7 homology domains. Science 275, 1927–1930 (1997) . 9072969 10.1126/science.275.5308.1927

[b42] Welch H. C. . P-Rex1, a PtdIns(3,4,5)P3- and Gbetagamma-regulated guanine-nucleotide exchange factor for Rac. Cell 108, 809–821 (2002) . 11955434 10.1016/s0092-8674(02)00663-3

[b43] Cancelas J. A. . Rac GTPases differentially integrate signals regulating hematopoietic stem cell localization. Nat. Med. 11, 886–891 (2005) . 16025125 10.1038/nm1274

[b44] Gu Y. . Hematopoietic cell regulation by Rac1 and Rac2 guanosine triphosphatases. Science 302, 445–449 (2003) . 14564009 10.1126/science.1088485

[b45] Gao Y., Dickerson J. B., Guo F., Zheng J. & Zheng Y. Rational design and characterization of a Rac GTPase-specific small molecule inhibitor. Proc. Natl Acad. Sci. USA 101, 7618–7623 (2004) . 15128949 10.1073/pnas.0307512101 PMC419655

[b46] Thomas E. K. . Rac guanosine triphosphatases represent integrating molecular therapeutic targets for BCR-ABL-induced myeloproliferative disease. Cancer Cell 12, 467–478 (2007) . 17996650 10.1016/j.ccr.2007.10.015

[b47] Schwartz S. . Feedback suppression of PI3Kalpha signaling in PTEN-mutated tumors is relieved by selective inhibition of PI3Kbeta. Cancer Cell 27, 109–122 (2015) . 25544636 10.1016/j.ccell.2014.11.008 PMC4293347

[b48] Berenjeno I. M. . Both p110alpha and p110beta isoforms of PI3K can modulate the impact of loss-of-function of the PTEN tumour suppressor. Biochem. J. 442, 151–159 (2012) . 22150431 10.1042/BJ20111741 PMC3268223

[b49] Weiner O. D. . A PtdInsP(3)- and Rho GTPase-mediated positive feedback loop regulates neutrophil polarity. Nat. Cell Biol. 4, 509–513 (2002) . 12080346 10.1038/ncb811 PMC2823287

[b50] Shirsat N. V., Pignolo R. J., Kreider B. L. & Rovera G. A member of the ras gene superfamily is expressed specifically in T, B and myeloid hemopoietic cells. Oncogene 5, 769–772 (1990) . 2189110

[b51] Yang F. C. . Rac and Cdc42 GTPases control hematopoietic stem cell shape, adhesion, migration, and mobilization. Proc. Natl Acad. Sci. USA 98, 5614–5618 (2001) . 11320224 10.1073/pnas.101546898 PMC33261

[b52] Mizukawa B. . Inhibition of Rac GTPase signaling and downstream prosurvival Bcl-2 proteins as combination targeted therapy in MLL-AF9 leukemia. Blood 118, 5235–5245 (2011) . 21940819 10.1182/blood-2011-04-351817 PMC3217406

[b53] Kalaitzidis D. . mTOR complex 1 plays critical roles in hematopoiesis and Pten-loss-evoked leukemogenesis. Cell Stem Cell 11, 429–439 (2012) . 22958934 10.1016/j.stem.2012.06.009 PMC3743253

[b54] Magee J. A. . Temporal changes in PTEN and mTORC2 regulation of hematopoietic stem cell self-renewal and leukemia suppression. Cell Stem Cell 11, 415–428 (2012) . 22958933 10.1016/j.stem.2012.05.026 PMC3447536

[b55] Saci A., Cantley L. C. & Carpenter C. L. Rac1 regulates the activity of mTORC1 and mTORC2 and controls cellular size. Mol. Cell. 42, 50–61 (2011) . 21474067 10.1016/j.molcel.2011.03.017 PMC3750737

[b56] Kalaitzidis D. & Neel B. G. Flow-cytometric phosphoprotein analysis reveals agonist and temporal differences in responses of murine hematopoietic stem/progenitor cells. PLoS ONE 3, e3776 (2008) . 19020663 10.1371/journal.pone.0003776 PMC2582484

