# Supplementary material for: A PI3K p110β–Rac signalling loop mediates Pten-loss-induced perturbation of haematopoiesis and leukaemogenesis
Source: Nat Commun. 2015 Oct 7;6:8501. doi: 10.1038/ncomms9501 (PMC4598950; doi:10.1038/ncomms9501)
Supplement: Supplementary Information — Supplementary Figures 1-10 [file ncomms9501-s1.pdf]

Supplementary Information:

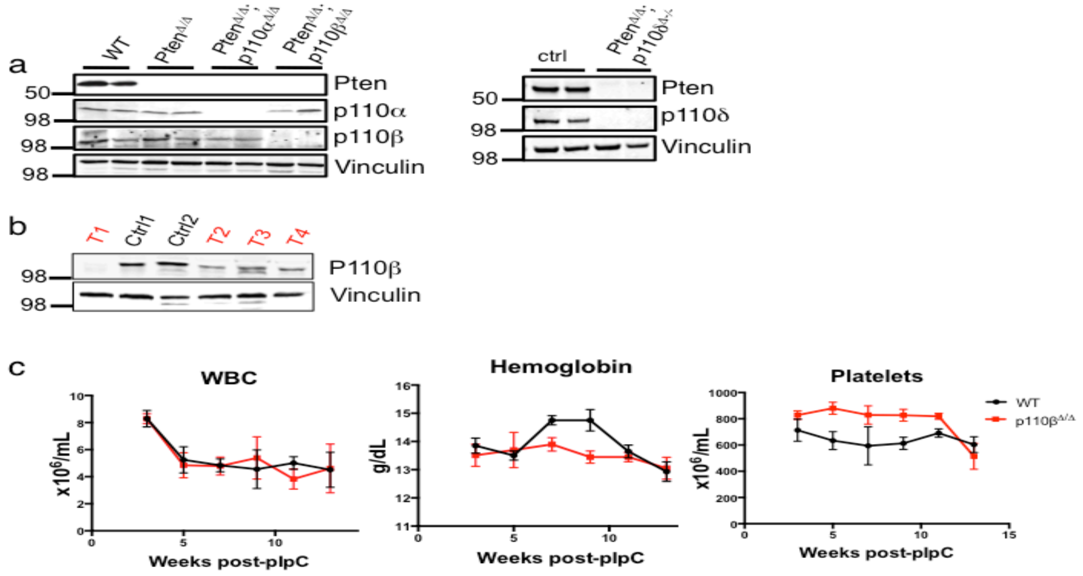

Supplementary Figure 1. Successful excision of genes from WBM lysates and survival of mice with different genotypes.

(a) The proper excision of Pten, p110α, p110α and p110δ was confirmed by western blotting for the respective isoform using spleens at 7 DPI.

(b) Western blot analysis of p110β in tumors (T2, T3 and T4) BM which arose in a pI-pC treated *Mx-1-cre+;Pten*$^{f/f}$*;p110β*$^{f/f}$ mouse (the three *Pten*$^{Δ/Δ}$*;p110□*$^{Δ/Δ}$ mice which developed MPN following pI-pC administration and died earlier). Note that these three BM cells express p110β protein, revealing that it arose from the residual, non-deleted p110β gene. For control, a *Pten*$^{Δ/Δ}$*;p110β*$^{Δ/Δ}$ BM (T1) which successfully has deleted P110β gene at the same time and p110β positive Ctrl1 and Ctrl2 BM are shown.

(c) *p110β*$^{f/f}$;Mx1-Cre mice were injected with pIpC 250□g x3 at 4 weeks post -natally. Peripheral blood counts were obtained at regular intervals. White blood cell, hemoglobin, and platelet counts from WT or p110β$^{Δ/Δ}$ mice□were monitored for over 15 weeks after pIpC excision (n=7-16 per group).

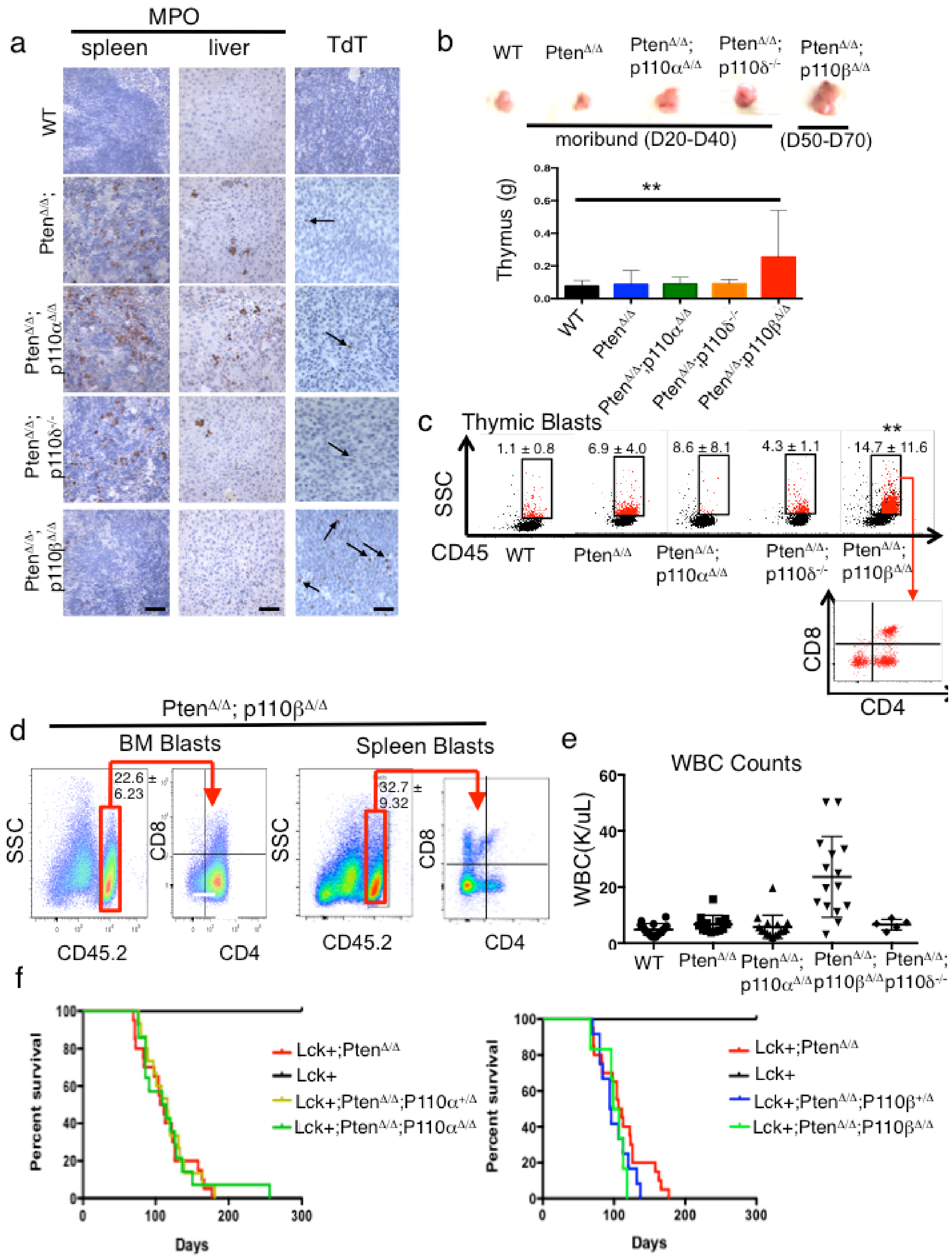

Supplementary Figure 2. Disease phenotypes of moribund animals.

(a) MPO staining in spleen and liver showing reduced myeloid infiltration in *Pten*$^{\Delta/\Delta}$*;p110*$\beta^{\Delta/\Delta}$ animals (left panels). Terminal deoxynucleotidyl transferase (TdT) staining in thymuses of moribund animals showing increased presence of lymphoid blast in the thymus of *Pten*$^{\Delta/\Delta}$*;p110*$\beta^{\Delta/\Delta}$ animals (right panels).

(b) Pictures of thymuses of moribund animals (top panel) showing increased thymus size in *Pten*$^{\Delta/\Delta}$*;p110*$\beta^{\Delta/\Delta}$ animals at the moribund stage (D20 and D40 for *Pten*$^{\Delta/\Delta}$, *Pten*$^{\Delta/\Delta}$*;p110*$\alpha^{\Delta/\Delta}$ and *Pten*$^{\Delta/\Delta}$*;p110*$\delta^{-/-}$, D5-D70 for *Pten*$^{\Delta/\Delta}$*;p110*$\beta^{\Delta/\Delta}$). Thymuses were isolated at the moribund stage and weighted (lower panel). n = 12 (ctrl), 18 (*Pten*$^{\Delta/\Delta}$), 15 (*Pten*$^{\Delta/\Delta}$*;p110*$\alpha^{\Delta/\Delta}$), 25 (*Pten*$^{\Delta/\Delta}$*;p110*$\beta^{\Delta/\Delta}$), and 6 (*Pten*$^{\Delta/\Delta}$*;p110*$\delta^{-/-}$).

(c) Blasts were identified based on cell size in flow cytometry and further characterized for CD4 and CD8 expression as shown in B. n = 2 (*Pten*$^{\Delta/\Delta}$*;p110*$\delta^{-/-}$) , 6 (*Pten*$^{\Delta/\Delta}$*;p110*$\beta^{\Delta/\Delta}$), 7 (*Pten*$^{\Delta/\Delta}$, *Pten*$^{\Delta/\Delta}$*;p110*$\alpha^{\Delta/\Delta}$), and 8 (ctrl).

(d) Flow cytometry showing leukemic blasts as in c. of *Pten*$^{\Delta/\Delta}$*;p110*$\beta^{\Delta/\Delta}$ in spleen and BM. (n=2-3 per group)

(e) WBC counts of each group at moribund stage. (n=10-12 per group, except *Pten*$^{\Delta/\Delta}$*;p110*$\delta^{-/-}$ *)(*n=6*)*

(f) Survival plot of *Lck+;Pten*$^{WT/WT}$*, Lck+;Pten*$^{-/-}$, *Lck+P110*$\alpha^{+/-}$*;Pten*$^{-/-}$ and *Lck+P110*$\alpha^{-/-}$*;Pten*$^{-/-}$ as well as survival plot of *Lck+;Pten*$^{WT/WT}$*, Lck+;Pten*$^{-/-}$, *Lck+P110*$\beta^{+/-}$*;Pten*$^{-/-}$ and *Lck+P110*$\beta^{-/-}$*;Pten*$^{-/-}$ mice mice showed loss of p110α or p110β does not have any effect on Lck-Cre-mediated Pten loss T-ALL (n=15).

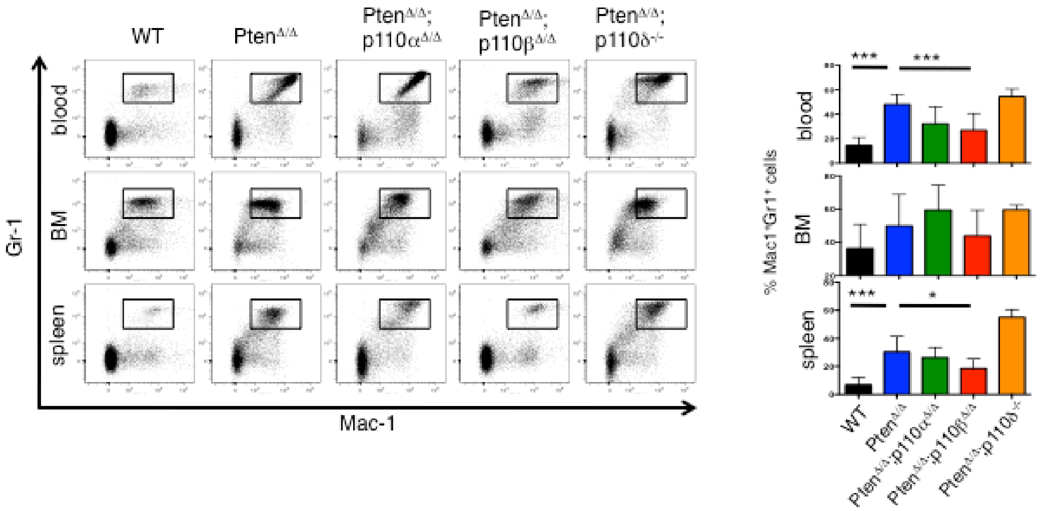

Supplementary Figure 3. Ablation of p110β blocked myeloid cells infiltration in peripheral blood, BM and spleen.

Representative flow cytometry plots of Mac1$^+$Gr1$^+$ cells in the blood, BM, and spleen (top panel). Samples were analyzed at 26 DPI. Bar graphs (lower panel) represent the mean ± SD of n = 9 (*Pten*$^{\Delta/\Delta}$*;p110*$\delta^{-/-}$), 10 (*Pten*$^{\Delta/\Delta}$*;p110*$\alpha^{\Delta/\Delta}$), 11 (ctrl), or 13 (*Pten*$^{\Delta/\Delta}$ and *Pten*$^{\Delta/\Delta}$*;p110*$\beta^{\Delta/\Delta}$).

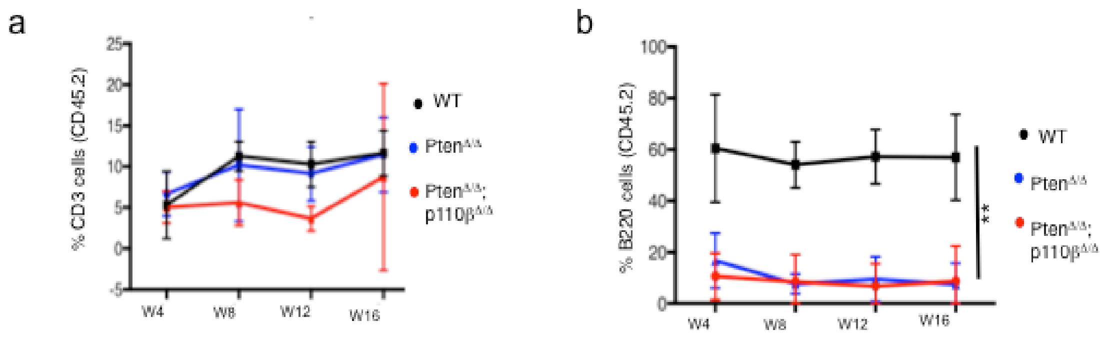

Supplementary Figure 4. T cell and B cell reconstitution potential in recipient mice.

(a) The T cell (by CD3 expression) reconstitution potential of the CD45.2^{+} cells was measured at regular intervals for 16 weeks. (n = 10 for all groups)

(b) The B cell (by B220 expression) reconstitution potential of the CD45.2^{+} cells was measured at regular intervals for 16 weeks. (n = 10 for all groups, **: $p < 0.001$). Two-way Anova test was applied to compare B cell potential.

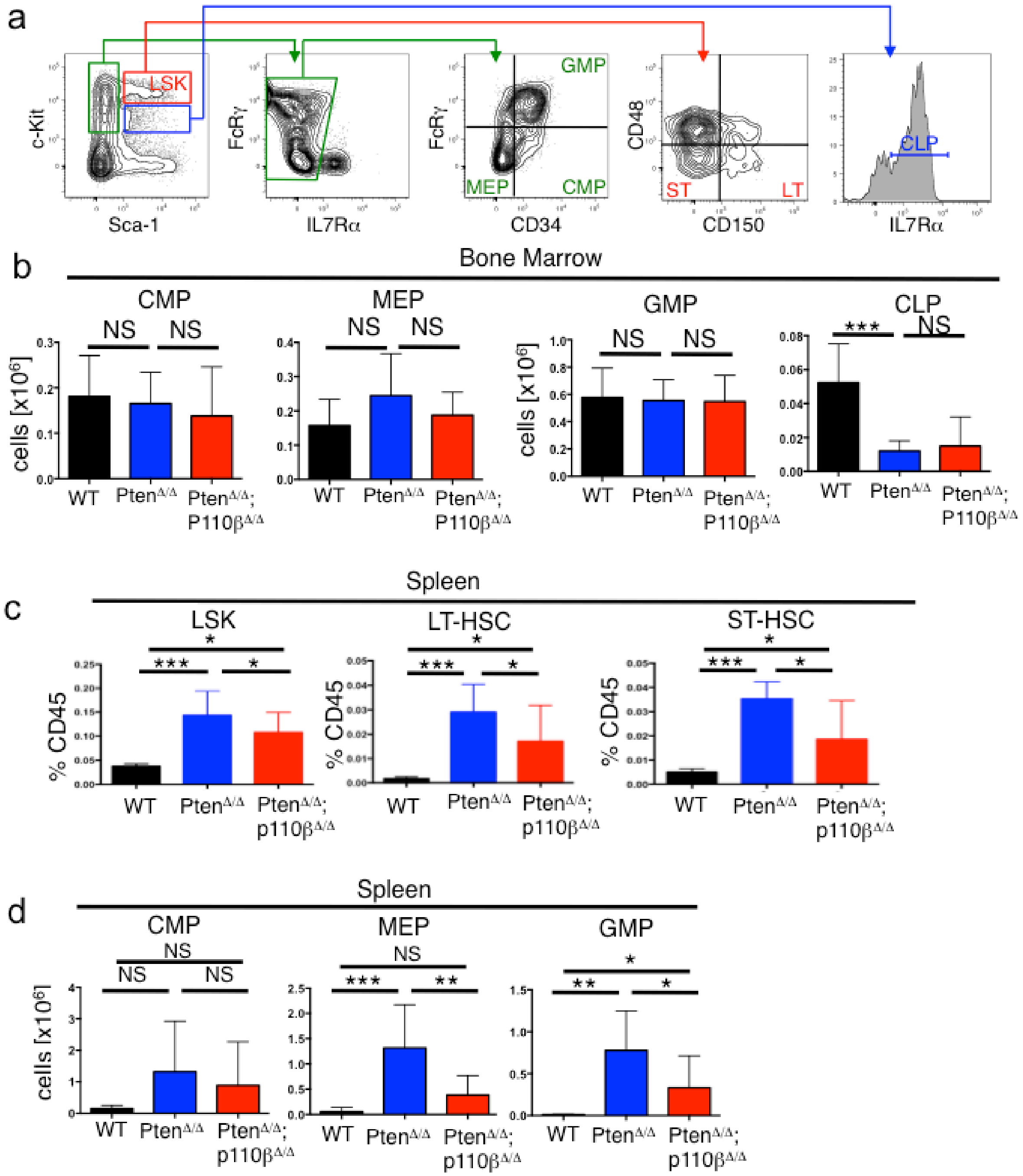

Supplementary Figure 5. Gating strategy to identify different cell populations and stem cells and progenitor frequencies.

(a) Gating strategy (top panels) to identify LSK cells (Lin$^-$Sca-1$^+$c-kit$^+$), ST-HSC (CD150$^-$CD48$^-$Lin$^-$ Sca-1$^+$c-kit$^+$), and LT-HSC (CD150$^+$CD48$^-$Lin$^-$ Sca-1$^+$c-kit$^+$) cells (all

red); MEP (FcRγ^{-}CD34^{-}IL7Rα^{-}Lin^{-}Sca-1^{-}c-kit^{+}), CMP (FcRγ^{-}CD34^{+}IL7Rα^{-}Lin^{-}Sca-1^{-}c-kit^{+}), GMP (FcRγ^{+}CD34^{+}IL7Rα^{-}Lin^{-}Sca-1^{-}c-kit^{+}) cells (all green); and CLP cells (IL7Rα^{+} Lin^{-} Sca-1^{+}c-kit^{low}) (blue).

(b) MEP (FcRγ^{-}CD34^{-}IL7Rα^{-}Lin^{-}Sca-1^{-}c-kit^{+}), CMP (FcRγ^{-}CD34^{+}IL7Rα^{-}Lin^{-}Sca-1^{-} c-kit^{+}), GMP (FcRγ^{+}CD34^{+}IL7Rα^{-}Lin^{-}Sca-1^{-}c-kit^{+}), and CLP cells (IL7Rα^{+} Lin^{-}Sca-1^{+}c-kit^{low}) cells were measured in the bone marrow of control, *Pten*$^{Δ/Δ}$, and *Pten*$^{Δ/Δ}$*;p110β*$^{Δ/Δ}$ animals and identified as shown in a. Numbers represent total cell counts per two tibia and two femurs (n = 7 (ctrl), 8 (*Pten*$^{Δ/Δ}$), and 11 (*Pten*$^{Δ/Δ}$*;p110β*$^{Δ/Δ}$)).

(c) Representative flow cytometry plots of control, *Pten*$^{Δ/Δ}$, and *Pten*$^{Δ/Δ}$*;p110β*$^{Δ/Δ}$ spleen cells showing the frequencies of LSK cells of CD45.2 (Lin^{-}Sca-1^{+}c-kit^{+}), ST-HSC (CD150^{-}CD48^{-}Lin^{-}Sca-1^{+}c-kit^{+}), and LT-HSC (CD150^{+}CD48^{-}Lin^{-}Sca-1^{+}c-kit^{+}) cells (left panels) measured at 26 DPI. (n = 7 (ctrl, *Pten*$^{Δ/Δ}$) and 10 (*Pten*$^{Δ/Δ}$*;p110β*$^{Δ/Δ}$)).

(d) MEP (FcRγ^{-}CD34^{-}IL7Rα^{-}Lin^{-}Sca-1^{-}c-kit^{+}), CMP (FcRγ^{-}CD34^{hi}IL7Rα^{-}Lin^{-}Sca-1^{-} c-kit^{+}), GMP (FcRγ^{+}CD34^{low}IL7Rα^{-}Lin^{-}Sca-1^{-}c-kit^{+}) were measured in spleens of ctrl, *Pten*$^{Δ/Δ}$, and *Pten*$^{Δ/Δ}$*;p110β*$^{Δ/Δ}$ animals. Numbers represent total cell counts per spleen (n = 7 (ctrl), 8 (*Pten*$^{Δ/Δ}$), and 11 (*Pten*$^{Δ/Δ}$*;p110β*$^{Δ/Δ}$)).

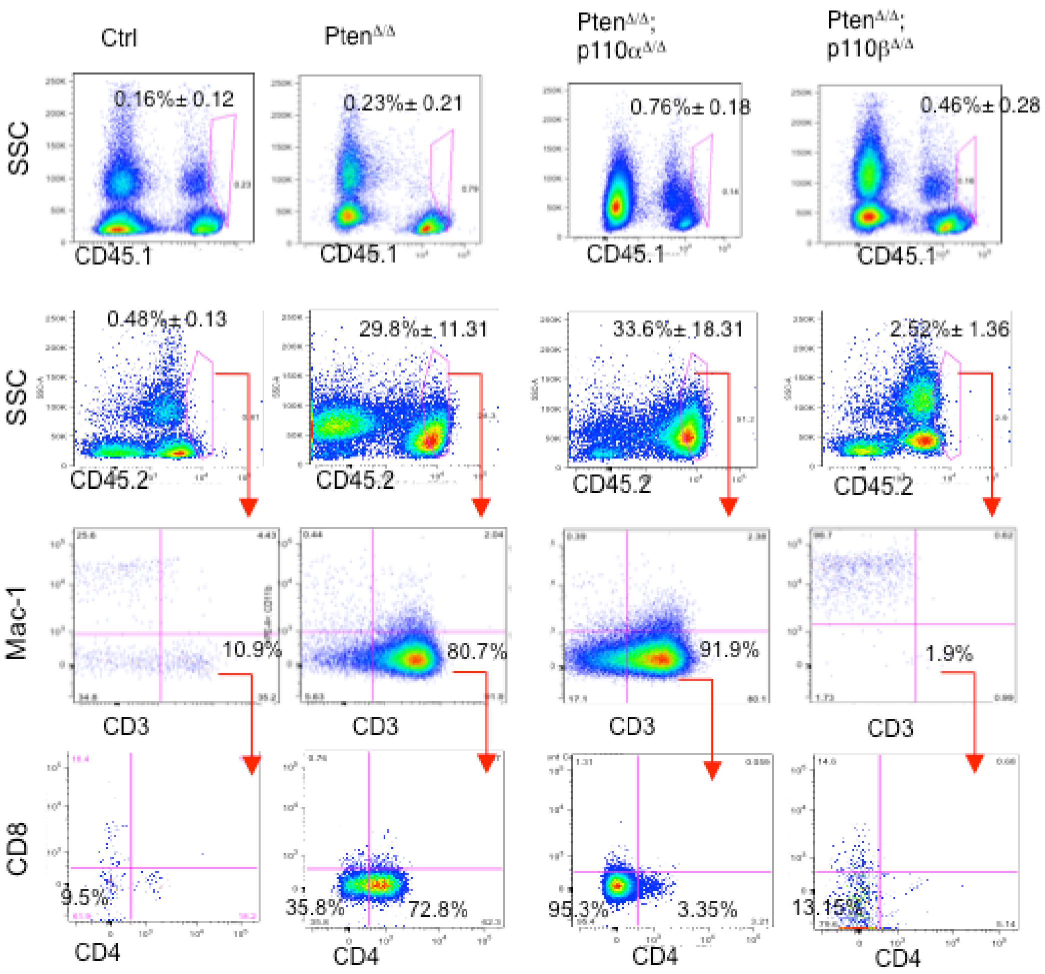

Supplementary Figure 6. Flow cytometry detection of leukemic blasts in the recipient mice.

Flow cytometry detection of leukemic blasts CD45.2 positive, CD45.1 negative, Mac1 negative and CD3+ at at week 20 (n=3-5 for each group)

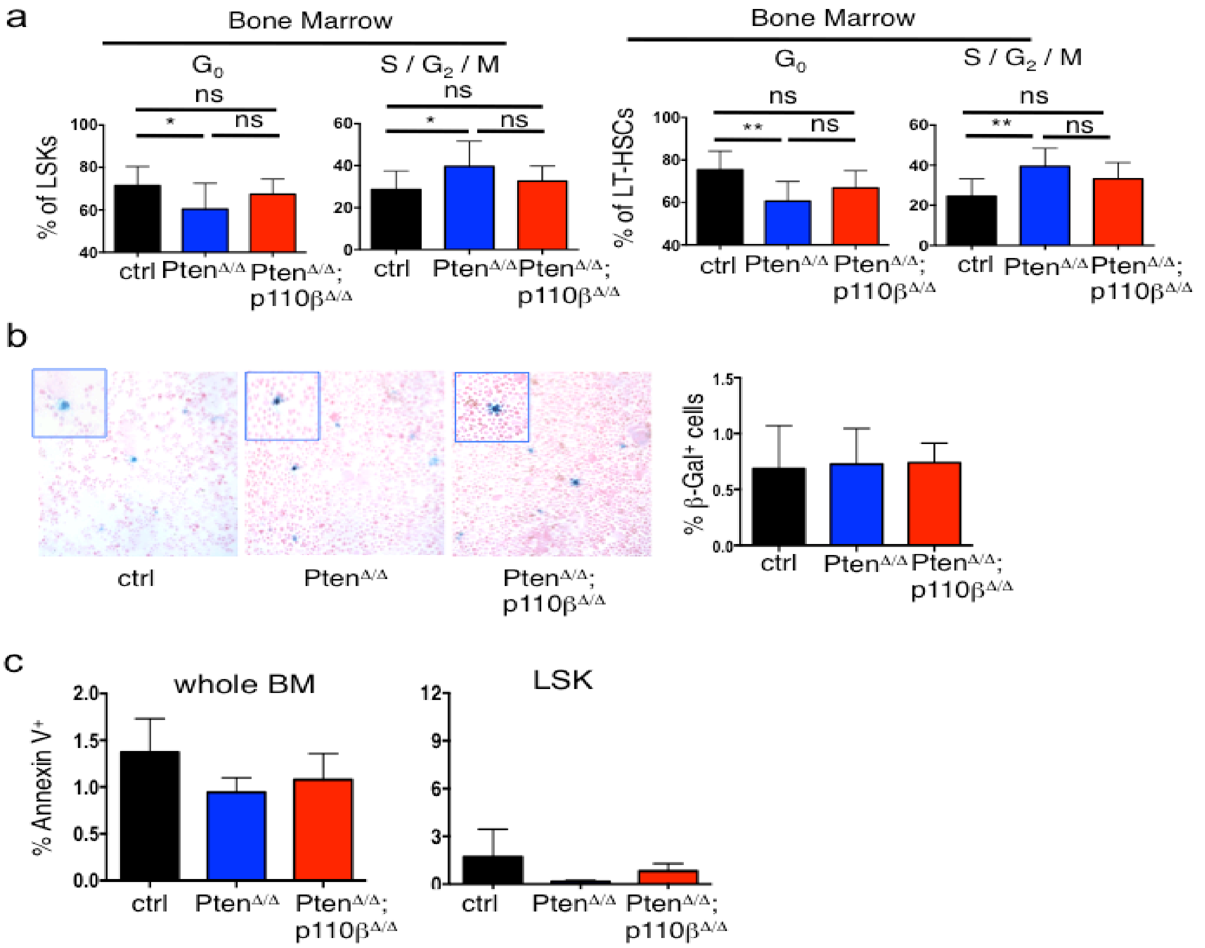

Supplementary Figure 7. No impact of p110β on cycling, senescence, or apoptosis of hematopoietic stem and progenitor cells in *Pten*$^{Δ/Δ}$ bone marrow.

(a) BM cells obtained on 7 DPI were stained with pyronin H and Hoechst and gated on LSK cells or LT-HSCs. (n = 3 for all groups).

(b) Senescence of cells was measured in BM by staining for β-gal activity (left panel). Mean ± SD is shown in right panel for n = 5 (ctrl) and 4 (*Pten*$^{Δ/Δ}$, and *Pten*$^{Δ/Δ}$*;p110β*$^{Δ/Δ}$).

(c) Annexin V staining was performed on bone marrow cells obtained 7 DPI. Cells were gated on whole bone marrow (left panel), or on LSK cells (right panel). No major change was observed in any of the groups (n = 5 (*Pten*$^{Δ/Δ}$*;p110β*$^{Δ/Δ}$) 4 (*Pten*$^{Δ/Δ}$).

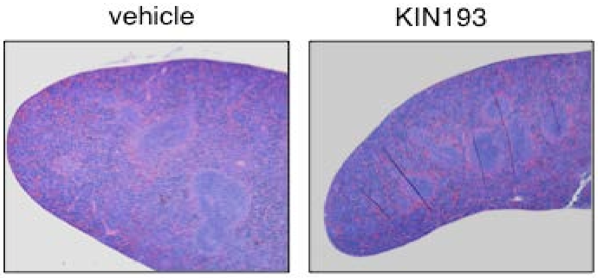

Supplementary Figure 8. p110β inhibitor KIN193 suppressed MPN disease in *Pten$^{\Delta/\Delta}$* *mice.*

H&E stained sections through the spleens (4x) showing reduced spleen size and intact spleen architecture at the moribund stage.

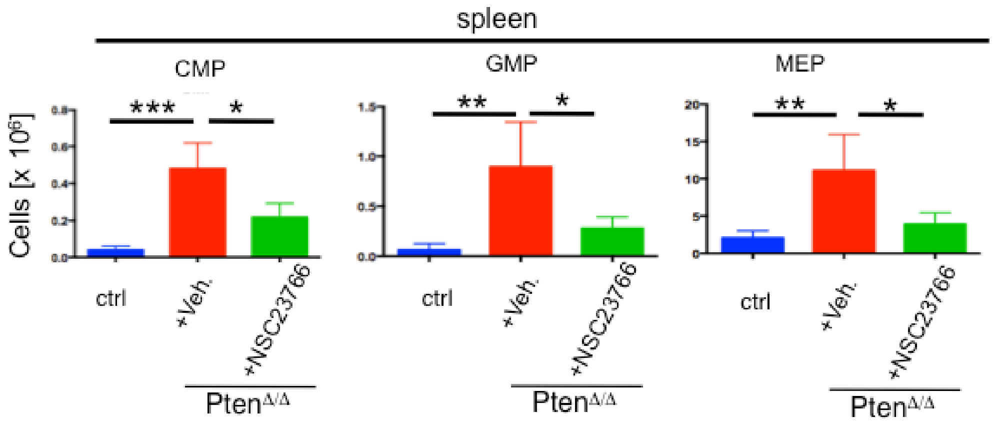

Supplementary Figure 9. Rac inhibitor NSC23766 suppressed progenitor cells in spleens of *Pten$^{Δ/Δ}$ mice.*

Representative flow cytometry quantifications of spleens of control, *Pten$^{Δ/Δ}$* animals treated with vehicle or NSC23766 at 17 DPI showing MEP (FcRγ^{-}CD34^{-}IL7Rα^{-}Lin^{-}Sca-1^{-}c-kit^{+}), CMP (FcRγ^{-}CD34^{+}IL7Rα^{-}Lin^{-}Sca-1^{-}c-kit^{+}), GMP (FcRγ^{+}CD34^{+}IL7Rα^{-}Lin^{-}Sca-1^{-}c-kit^{+}) were measured in spleens of the *Pten$^{Δ/Δ}$* animals. *: $p < 0.05$; **: $p < 0.001$; ***: $p<0.0001$. Two-way Anova test was applied to calculate the flow cytometry quantifications.

For Figure 5e.

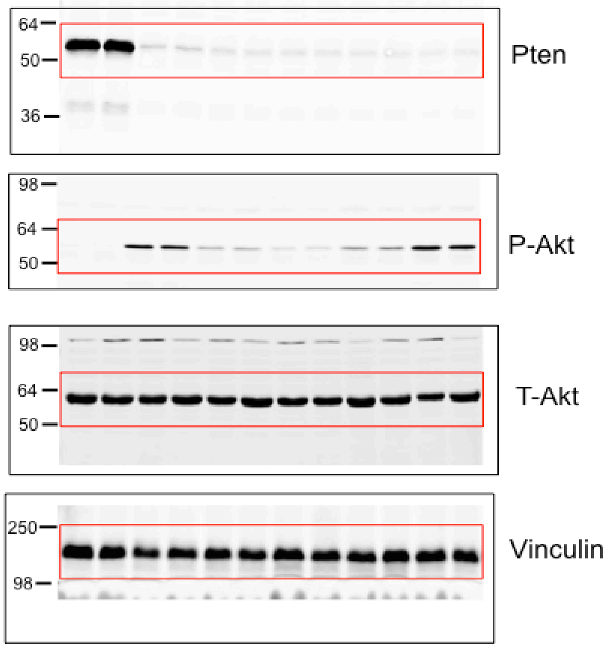

For Figure 6a.

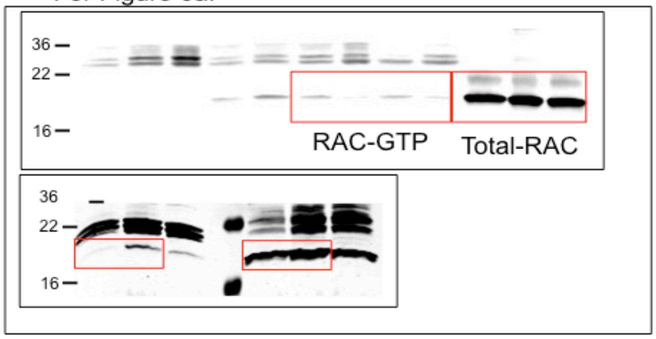

For Figure 6b.

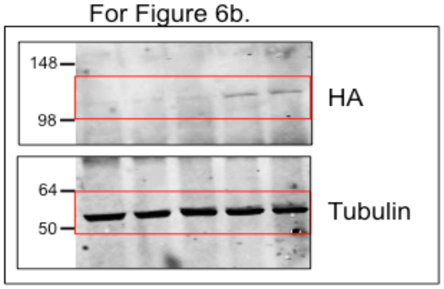

For Figure 6d.

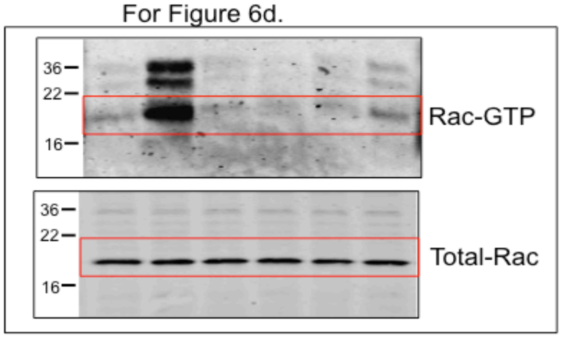

For Figure 7e.

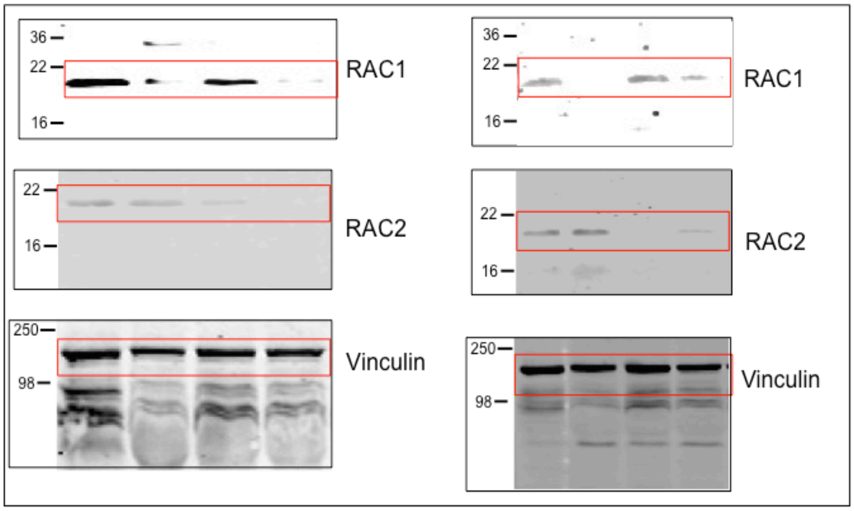

Supplementary Figure 10.

Original Western blotting images of the key immunoblotting experiments.
